# Tyrosine kinase LYN restricts the replication and virulence of influenza A virus by directly phosphorylating viral nucleoprotein

**DOI:** 10.1128/mbio.03759-25

**Published:** 2026-03-30

**Authors:** Junwen Liu, Jiaxin Huang, Qian Wang, Yanli Wei, Lebin Han, Xin Li, Caoqi Lei, Guohua Deng, Hualan Chen, Qiyun Zhu, Shuai Xu

**Affiliations:** 1 State Key Laboratory of Animal Disease Control and Prevention, College of Veterinary Medicine, Lanzhou University, Lanzhou Veterinary Research Institute, Chinese Academy of Agricultural Sciences 111658 , Lanzhou, People's Republic of China; 2 School of Basic Medical Sciences, Lanzhou University 426140, Lanzhou, People's Republic of China; 3 State Key Laboratory of Animal Disease Control and Prevention, Harbin Veterinary Research Institute, Chinese Academy of Agricultural Sciences 111613 , Harbin, People's Republic of China; 4 Gansu Province Research Center for Basic Disciplines of Pathogen Biology, Lanzhou, People's Republic of China; Boston University Chobanian & Avedisian School of Medicine, Boston, Massachusetts, USA

**Keywords:** influenza A virus, LYN, tyrosine kinase, phosphorylation, nucleoprotein

## Abstract

The continuous antigenic shift and drift of influenza A virus (IAV) result in the emergence of novel strains and drug resistance. Accordingly, it is urgent to develop novel antiviral drugs that target host factors. Here, we revealed that tyrosine kinase LYN (Lck/Yes-related novel protein tyrosine kinase) interacts directly with nucleoprotein (NP) and reduces the replication and virulence of IAV *in vitro* and *in vivo*, in a kinase-dependent manner. By directly catalyzing the tyrosine phosphorylation of NP at Y10/40/97 sites, LYN impairs the interaction of NP with viral RNA and polymerase proteins, as well as the oligomerization of NP, and thus negatively modulates the assembly of the viral ribonucleoprotein complex. The NP^Y10/40/97F^ mutation significantly increases the pathogenicity of IAV in mice. Furthermore, the LYN-specific agonist MLR-1023 showed promising therapeutic effects against IAV. Collectively, our findings suggest that LYN is a novel host kinase restricting IAV replication, and a promising target for anti-influenza drug development.

**IMPORTANCE:**

The nucleoprotein (NP) of influenza A virus (IAV) is a highly conserved, multifunctional, and the most abundant viral protein in infected cells, which makes NP a promising target for the development of anti-influenza drugs. Phosphorylation plays a crucial role in the stability and functionality of NP. However, few kinases have been identified that directly phosphorylate NP. In this study, LYN (Lck/Yes-related novel protein tyrosine kinase) was identified as a novel kinase that directly catalyzes the tyrosine phosphorylation of IAV NP and thus restricts the replication and virulence of IAV. The LYN-specific agonist MLR-1023 exhibited promising protective efficacy against lethal IAV infection. Our findings discovered LYN and LYN-agonist as potential drug candidates targeting IAV NP, which will facilitate the development of host-directed antiviral therapies.

## INTRODUCTION

Influenza A virus (IAV) is a widespread zoonotic pathogen that infects humans and various other animal species. To date, four pandemics have been caused by IAVs of the H1N1, H2N2, and H3N2 subtypes, resulting in over 50 million fatalities (1, 2). Furthermore, H5 and H7 avian influenza viruses have infected more than 2,600 humans and caused more than 1,000 deaths, in addition to numerous disease outbreaks in birds and other species (3). Since March 2024, the 2.3.4.4b clade of the H5N1 highly pathogenic avian influenza virus has triggered unprecedented outbreaks in cattle across the United States and caused at least 70 human infections, with six severe cases (4, 5).

The IAV genome consists of eight single-stranded viral RNA (vRNA) segments, which are wrapped by nucleoprotein (NP) and packaged together into separate viral ribonucleoprotein complexes (vRNPs) (6). The vRNPs of IAV are the basic functional unit of viral replication and transcription (7). NP is the major component of vRNP and gets extensively phosphorylated at various serine, threonine, and tyrosine residues (8). The phosphorylation regulates multiple functions of NP, such as the oligomerization, nuclear localization, assembly of nascent vRNP complexes, and ultimately viral polymerase activity (9). Several phosphorylated residues in NP have been identified, including S9, Y10, S165, T188, Y296, S407, and S486 (8, 10–12). Yet, few reports have focused on the kinases that directly catalyze the phosphorylation of IAV NP.

Src family kinases (SFKs) are the largest non-receptor tyrosine kinase family, which are activated by cell-surface specific receptors (13). Following activation, SFKs phosphorylate and activate multiple downstream signaling factors, such as NF-κB, MAPK, STAT, and PI3K, to regulate chemotaxis, survival, proliferation, and differentiation (14–16). LYN (Lck/Yes-related novel protein tyrosine kinase) is 512 amino acids in length, containing a unique domain (amino acids 1–62, UD), Src homology 3 domain (amino acids 63–123, SH3), Src homology 2 domain (amino acids 124–227, SH2), and a kinase domain (amino acids 247–501, KD) (17). LYN is mainly expressed in myeloid cells, B cells, and hematopoietic cells and participates in cell proliferation, differentiation, apoptosis, migration, inflammation, and tumorigenesis (18–21). Until now, the role of LYN in the life cycle of the virus has not been fully investigated.

Here, we sought to identify host factors that were involved in the life cycle of IAV and identified the tyrosine kinase LYN as a novel negative regulator of IAV replication. LYN specifically interacted with and phosphorylated NP to suppress IAV replication. In addition, the LYN agonist MLR-1023 attenuated IAV replication *in vivo* and *in vitro*. LYN might be a potential drug target for anti-influenza therapy.

## RESULTS

### LYN negatively regulates IAV replication *in vitro*

**Fig 1:**
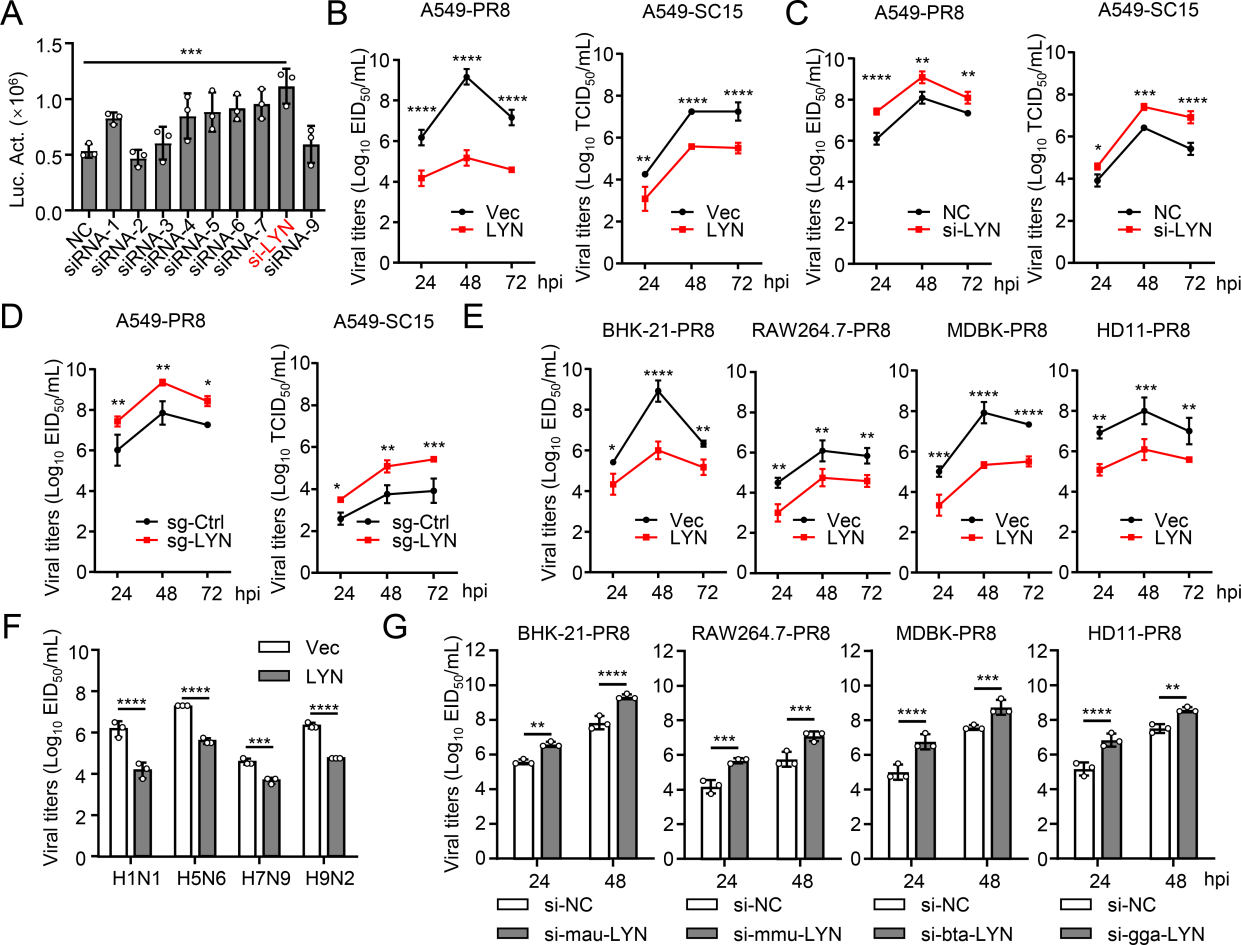
Identification of LYN as a negative regulator of IAV replication *in vitro*. (**A**) A549 cells were transfected with the indicated siRNAs for 24 h before infection with SC15-Nluc. Supernatants were analyzed at 24 h post-infection by use of a luciferase assay. (**B**) A549 cells were transfected with either the LYN expression plasmid or empty vector (Vec) for 24 h before infection with PR8 virus (H1N1, multiplicity of infection [MOI] = 0.01) or SC15 virus (H5N6, MOI = 0.01). Supernatants were collected at the indicated times post-infection for viral titration. (**C**) A549 cells were transfected with either the LYN siRNA or scrambled siRNA (NC) for 24 h before infection with PR8 virus (MOI = 0.01) or SC15 virus (MOI = 0.01). Supernatants were collected at the indicated times post-infection for viral titration. (**D**) The LYN-deficient (sg-LYN) or control (sg-Ctrl) A549 cells were infected with PR8 virus (MOI = 0.01) or SC15 virus (MOI = 0.01). Supernatants were collected at the indicated times post-infection for viral titration. (**E**) BHK-21, RAW264.7, MDBK, and HD11 cells were transfected with either the LYN expression plasmid or Vec for 24 h before infection with PR8 virus (MOI = 0.01). Supernatants were collected at the indicated times post-infection for the EID_50_ assay. (**F**) A549 cells were transfected with either the LYN expression plasmid or Vec for 24 h before infection with H1N1, H5N6, H7N9, or H9N2 virus (MOI = 0.01) for 24 h, and the supernatants were collected for the EID_50_ assay. (**G**) BHK-21, RAW264.7, MDBK, and HD11 cells were transfected with either si-NC or si-RNA targeting the species-specific LYN gene, respectively. At 24 h post-transfection, the cells were infected with PR8 virus at the MOI of 0.01 at the indicated times, and the supernatants containing viral particles were collected for the EID_50_ assay. Statistical significance was determined by a one-way analysis of variance (ANOVA) in panel A, or two-way ANOVA in panels B–G. The data represent three independent experiments (mean ± SD, *n* = 3).

To identify host factors, especially kinases, that participate in the life cycle of IAV by interacting with NP, we generated human embryonic kidney 293T cells that stably expressed NP tagged with the S protein, a Flag, and the streptavidin-binding peptide (SFB). By performing affinity-purified mass spectrometry, we identified a list of host factors that interacted with NP, including several kinases (Table S1). Using a reporter virus (SC15-Nluc) and an RNA interference library, 10 host kinases that interacted with NP were found to affect the replication of IAV (Fig. 1A). Among them, LYN was the most effective negative regulator of IAV replication. Using wild-type viruses, we found that overexpression of LYN reduced the replication of PR8 (H1N1) and SC15 (H5N6) viruses, whereas knockdown or deficiency of LYN significantly enhanced viral replication (Fig. 1B through D; Fig. S1A through C). In addition, LYN derived from human inhibited IAV replication in cells derived from different species (BHK-21 from hamster, RAW264.7 from mouse, MDBK from cattle, and HD11 from chicken) (Fig. 1E; Fig. S1D). Furthermore, LYN inhibited the replication of different subtypes of IAVs (Fig. 1F). Using species-specific siRNA to interfere with the expression of LYN in cells derived from different species, it was apparent that knockdown of LYN significantly promoted the replication of IAV in cells from different species (Fig. 1G; Fig. S1E). These results indicate that LYN is a conserved negative regulator of IAVs.

### LYN decreases the replication and pathogenicity of IAV *in vivo*

**Fig 2:**
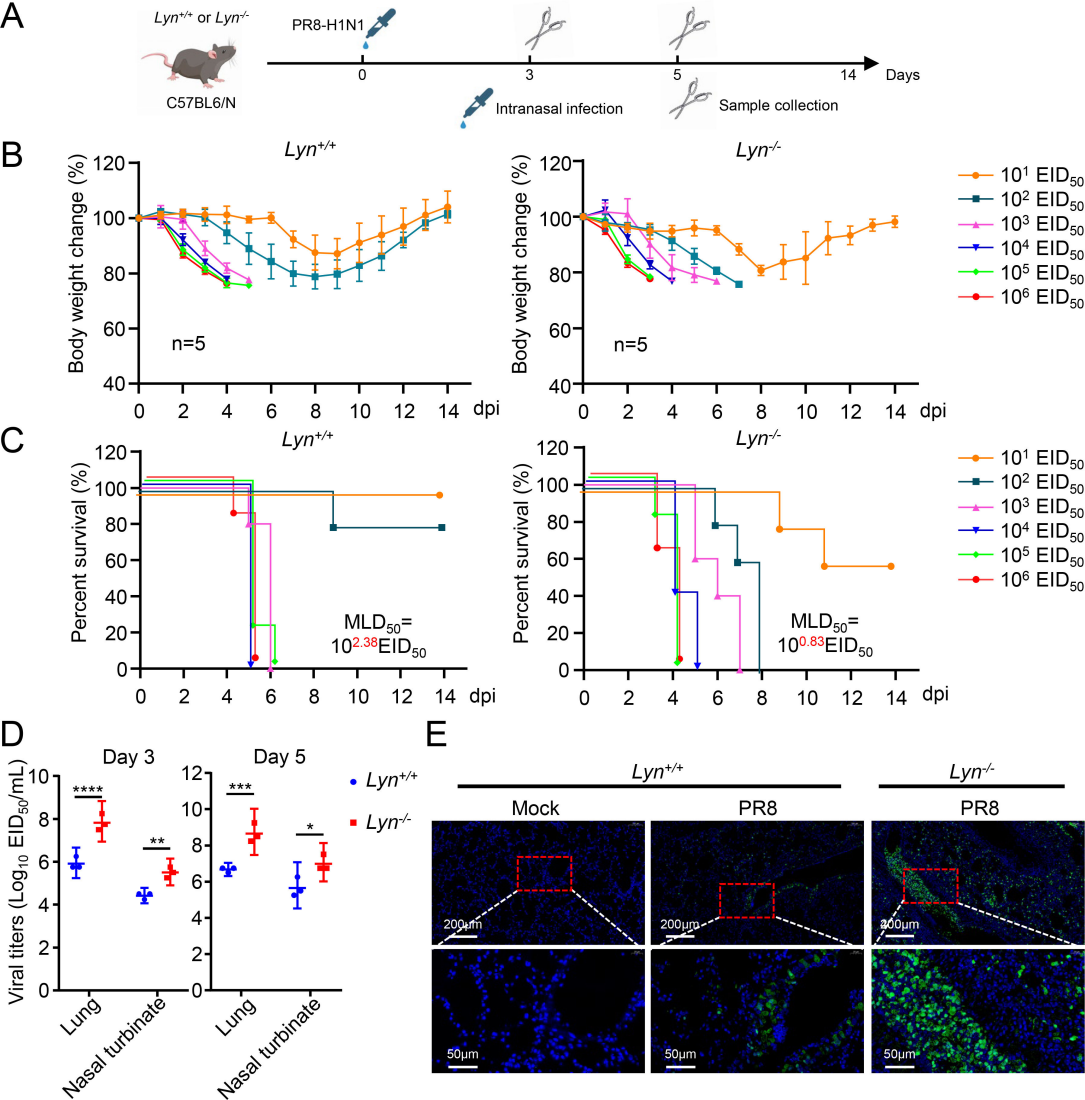
Identification of LYN as a negative regulator of IAV replication *in vivo*. (**A**) Diagram of experimental procedures in mice. Groups of 5 (*n* = 5) wild-type C57BL/6N (*Lyn^+/+^*) mice and *Lyn^−/−^* mice were intranasally inoculated with 10-fold serial dilutions containing 10^1^ to 10^6^ EID_50_ of PR8 virus. Changes in body weight (**B**) and survival (**C**) were monitored daily for 14 days after virus challenge. The MLD_50_ was calculated by using the method of Reed and Muench. (**D**) *Lyn^+/+^* and *Lyn^−/−^* mice were intranasally infected with PR8 (10^4^ EID_50_) virus and euthanized on days 3 and 5 post-inoculation (dpi). The lungs and nasal turbinate were collected for the EID_50_ assay. Statistical significance was determined by a two-way ANOVA. (**E**) Immunofluorescent staining of lung sections collected from *Lyn^+/+^* and *Lyn^−/−^* mice at 3 dpi with PR8 virus. The viral NP protein was stained green, and the nucleus was stained blue. *P* value was <0.05(*), <0.01(**), <0.001(***), and <0.0001(****).

To investigate the effect of LYN on the replication and pathogenicity of IAV *in vivo*, LYN knockout (*Lyn^–/–^*) mice were generated and used to determine the MLD_50_ (50% mouse lethal dose) of the PR8 virus (Fig. 2A). Bodyweight changes and survival rates indicated that the PR8 virus causes greater weight loss in *Lyn^–/–^* mice than in *Lyn^+/+^* mice, and the virulence of the PR8 virus in *Lyn^–/–^* mice (MLD_50_ = 10^0.83^ EID_50_) was 35-fold higher than that in *Lyn^+/+^* mice (MLD_50_ = 10^2.38^ EID_50_) (Fig. 2B and C). In addition, the virus load in the lung and nasal turbinate of *Lyn^–/–^* mice was significantly higher than that in *Lyn^+/+^* mice (Fig. 2D). An immunofluorescence assay showed that stronger NP antigen signals were detected in the lungs of virus-infected *Lyn^–/–^* mice compared with *Lyn^+/+^* mice (Fig. 2E; Fig. S2A). Similarly, more severe lung lesions were observed in virus-infected *Lyn^–/–^* mice compared with *Lyn^+/+^* mice (Fig. S2B and C). Collectively, these results demonstrate that LYN attenuates the replication and virulence of the PR8 virus *in vivo*.

### LYN specifically interacts with the NP protein of IAV

**Fig 3:**
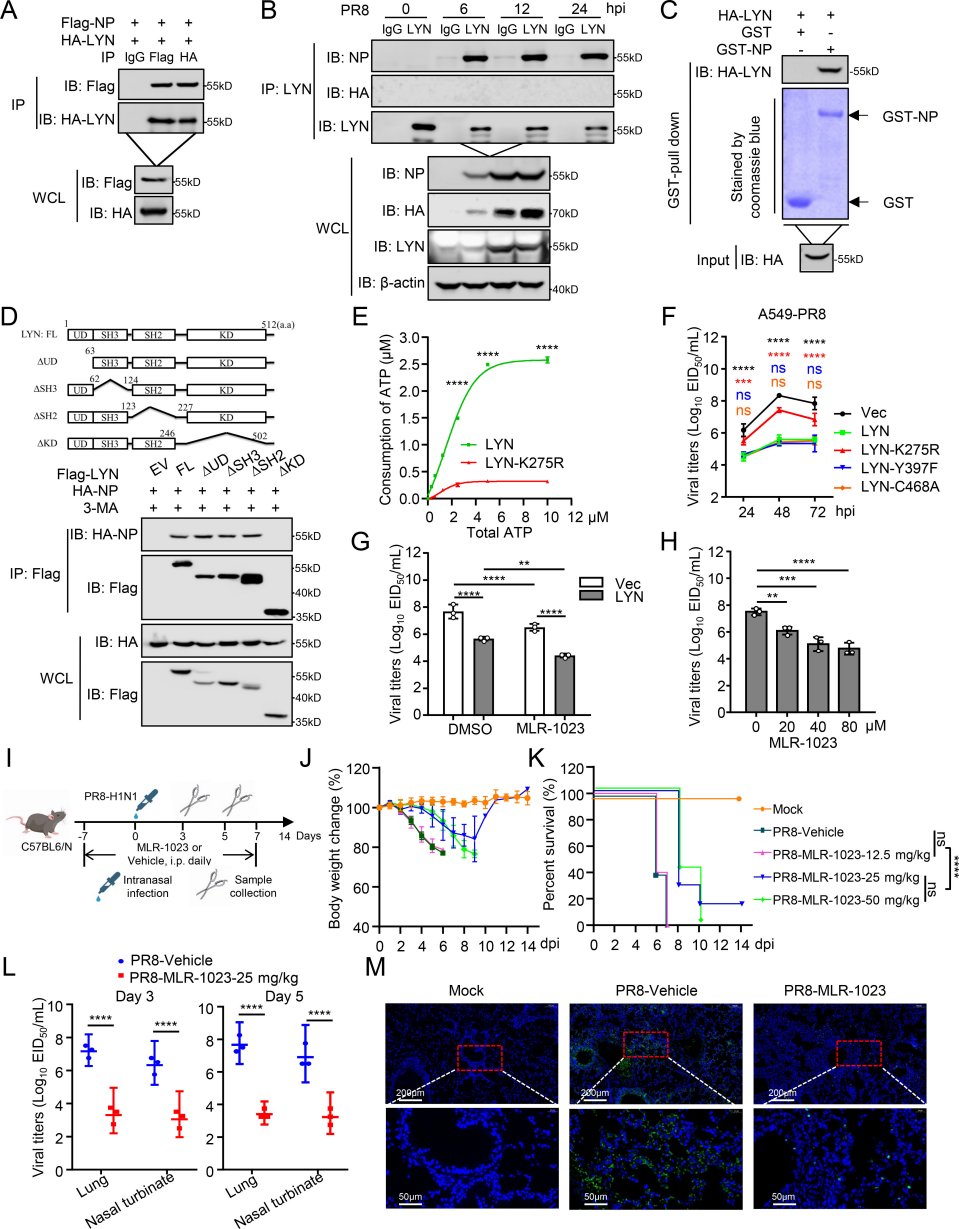
LYN restricts IAV replication through the kinase activity of LYN. (**A**) HEK293T cells were transfected with HA-LYN together with Flag-NP for 24 h before being IPed with anti-Flag or anti-HA antibody. (**B**) HEK293T cells were infected with PR8 virus and the cell lysate was co-IPed with anti-LYN antibody. (**C**) Purified GST-NP was used to pulldown transiently expressed HA-LYN, then analyzed by western blotting. (**D**) Domain mapping of LYN. HEK293T cells were transfected with HA-NP together with Flag-tagged LYN and LYN truncations for 24 h. The cells were then treated with 3-MA for 6 h before being co-IPed with anti-Flag antibody. (**E**) The kinase activity of purified LYN and LYN-K275R was detected using the kinase activity assay kit. (**F**) A549 cells were transfected with the expression plasmid of LYN, LYN mutants, or Vec for 24 h before being infected with PR8 virus (MOI = 0.01). Supernatants were collected at the indicated times post-infection for the EID_50_ assay. The significant difference between each group and wild-type LYN was labeled in indicated colors. (**G**) A549 cells were transfected with either the LYN expression plasmid or Vec for 24 h. Then, the cells were infected with PR8 virus (MOI = 0.01) and treated with MLR-1023 (20 μM) or DMSO. At 48 h post-infection, the supernatants were collected for the EID_50_ assay. (**H**) A549 cells were infected with PR8 virus (MOI = 0.01) and treated with MLR-1023 (0/20/40/80 μM). At 48 h post-infection, the supernatants were collected for the EID_50_ assay. (**I**) Flowchart illustrates the effect of MLR-1023 on IAV infection in the mouse model. Groups of 11 female C57BL/6N mice were infected with 10^5^ EID_50_ of PR8 virus. MLR-1023 at different concentrations was injected intraperitoneally from 7 days before challenge and administered for 14 days, once daily. Changes in body weight (**J**) and survival (**K**) were monitored for 14 days after virus challenge. On days 3 and 5 post-inoculation, three mice in each group were euthanized, and their lungs and nasal turbinate were collected for the EID_50_ assay. (**L**) Partial lung tissues collected on day 3 post-inoculation were subjected to immunofluorescence. (**M**) The viral NP protein was stained green, and the nucleus was stained blue. Statistical significance was determined by a one-way ANOVA in panels H and K, or a two-way ANOVA in panels E, F, G, and L. The data represent three independent experiments (mean ± SD, *n* = 3). *P* value was <0.01(**), <0.001(***), and <0.0001(****), “ns” indicates no significant difference.

Given that LYN was pulled down by NP in mass spectrometry, we next confirmed the interaction of LYN with NP. Coimmunoprecipitation (co-IP) results confirmed that Flag-tagged NP interacts with HA-tagged LYN, and the interaction between NP and LYN was independent of viral RNA (Fig. 3A; Fig. S3A). Following IAV infection, endogenous LYN associated with NP in virus-infected cells (Fig. 3B). A glutathione *S*-transferase (GST) pulldown assay demonstrated that LYN directly interacts with NP *in vitro* (Fig. 3C; Fig. S3C and D). In addition, LYN was diffusely distributed in cells without IAV infection, whereas LYN congregated and co-localized with NP after IAV infection (Fig. S3B). These results suggest that LYN specifically interacts with NP.

### The kinase activity of LYN is essential to restrict IAV replication

LYN is a tyrosine kinase, containing a UD domain, SH3 domain, SH2 domain, and a KD domain. Co-IP experiments indicated that the KD domain of LYN is required for the interaction with NP (Fig. 3D). Previous studies suggested that the K275/Y397/C468 residues are key sites for the kinase activity of LYN, and that the K275R substitution completely blocks the kinase activity of LYN, whereas Y397F and C468A partially reduce the kinase activity of LYN (22–24). As verification, our kinase assay confirmed that the K275R mutation significantly reduced the kinase activity of LYN (Fig. 3E). The viral dynamics indicated that wild-type LYN reduced the replication of IAV, the LYN-Y397F and LYN-C468A showed no difference with wild-type LYN, but the K275R mutation reduced the restrictive effect of LYN on IAV replication in cells (Fig. 3F; Fig. S3E).

Next, we used the commercially available LYN agonist MLR-1023 and inhibitors (Bafetinib and LYN-IN-1) to test the impact of LYN kinase activity on IAV replication in cells (25). MLR-1023 [Tolimidone; 2(1*H*)-pyrimidinone,5-(3-methylphenoxy)] is a direct, selective, allosteric activator of LYN (26). The CCK8 assay showed that 80 µM MLR-1023 had no effect on cell viability (Fig. S3F), and 20 μM MLR-1023 was sufficient to inhibit IAV replication and enhance the inhibitory effect of LYN on IAV replication (Fig. 3G). In addition, MLR-1023 decreased the replication of IAV in a dose-dependent manner (Fig. 3H). Correspondingly, treatment with LYN inhibitors (20 μM Bafetinib or 20 μM LYN-IN-1) blocked the restrictive effect of LYN on IAV replication (Fig. S3G).

Furthermore, we tested the effect of the agonist and inhibitors of LYN on the replication of IAV in a mouse model (Fig. 3I). MLR-1023 increased the self-phosphorylation of LYN (Fig. S3H). MLR-1023 administration (25 and 50 mg/kg/day) reduced the weight loss and mortality induced by IAV infection compared to vehicle controls (Fig. 3I through K). Likewise, the virus load of the nasal turbinate and lungs from MLR-1023-treated (25 mg/kg/day) mice was significantly lower than that in the control (Fig. 3L). Less NP antigen signal and tissue lesions were observed in MLR-1023-treated mice, compared with vehicle-treated mice (Fig. 3M; Fig. S3I and J). Collectively, these results indicate that LYN restricts IAV replication through its kinase activity.

### LYN directly phosphorylates NP at tyrosine 10/40/97

**Fig 4:**
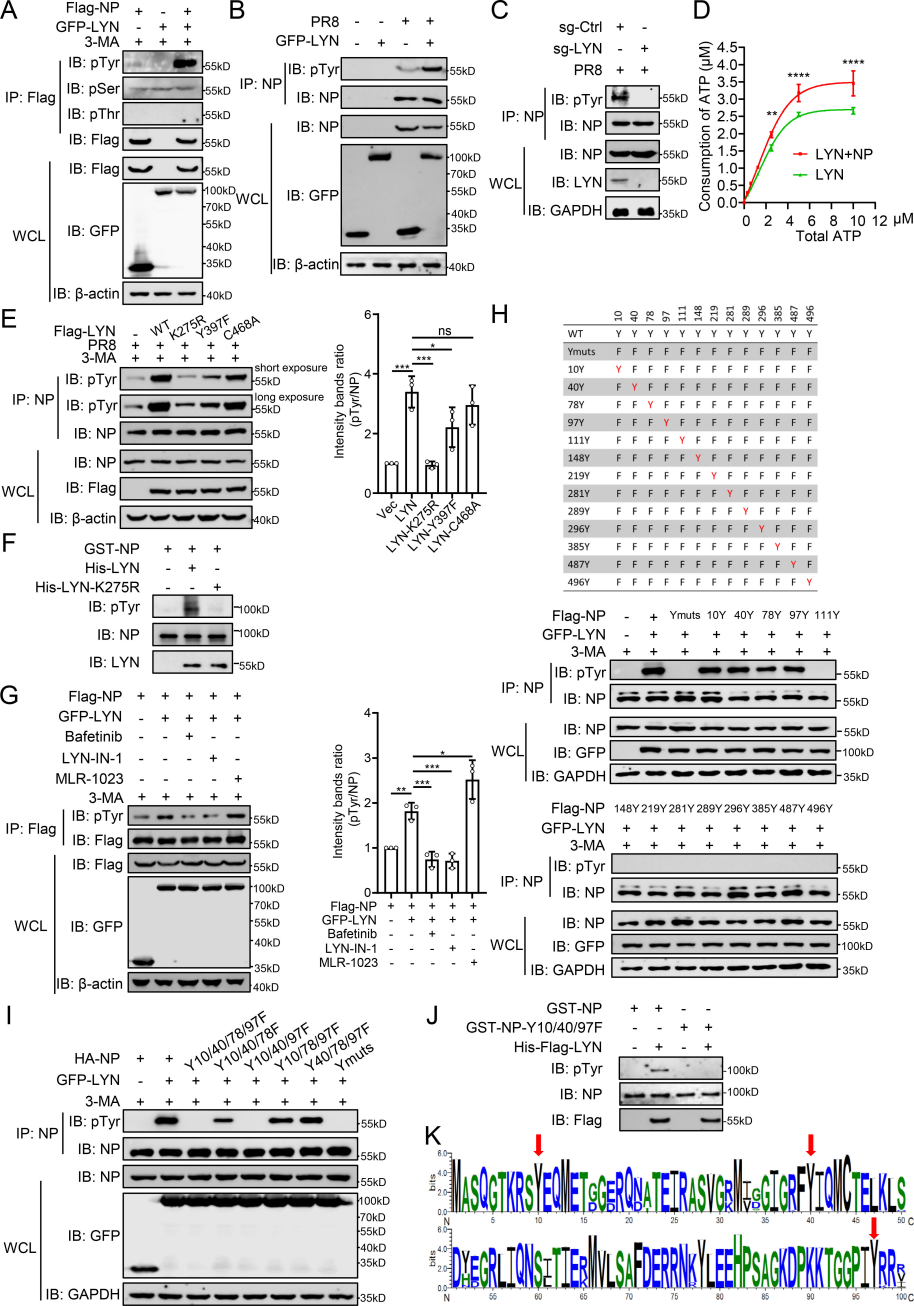
LYN directly and specifically catalyzes the tyrosine phosphorylation of NP. (**A**) HEK293T cells were transfected with GFP-LYN together with Flag-NP for 24 h before being co-IPed with anti-Flag antibody. (**B**) HEK293T cells were transfected with GFP-LYN for 24 h. Then, the cells were infected with PR8 virus and the cell lysate was co-IPed with anti-NP antibody. (**C**) The LYN-deficient (sg-LYN) or control (sg-Ctrl) A549 cells were infected with PR8 virus and the cell lysate was co-IPed with anti-NP antibody. (**D**) The kinase activity of purified LYN, with or without NP. (**E**) HEK293T cells were transfected with LYN or LYN mutants for 24 h. Then, the cells were infected with the PR8 virus, and the cell lysate was co-IPed with anti-NP antibody. The intensity ratio of phosphorylated NP normalized to NP in IP samples was determined using ImageJ. (**F**) Purified His-LYN or His-LYN (K275R) was subjected to an *in vitro* kinase assay with purified GST-NP. The intensity ratio of phosphorylated NP normalized to Flag-NP in IP samples was determined using ImageJ. (**G**) HEK293T cells were transfected with GFP-LYN and Flag-NP for 24 h, with or without treatment with MLR-1023 (20 μM), Bafetinib (20 μM), or LYN-IN-1 (20 μM). Then, the cells were treated with 3-MA for 6 h before being co-IPed with anti-Flag antibody. (**H and I**) Mutants of NP with replacement of various tyrosine residues (red “Y”), HEK293T cells were transfected with GFP-LYN together with NP or NP mutants for 24 h. The cells were then treated with 3-MA for 6 h before being co-IPed with anti-NP antibody. (**J**) Transiently expressed NP or NP mutants were subjected to an *in vitro* kinase assay with purified His-LYN. (**K**) NP amino acid sequence of all subtypes of IAVs. NP sequences were downloaded from GenBank using the “collapse identical sequences” option. Logos were generated using the WebLogo3 online tool (weblogo.threeplusone.com). The data represent three independent experiments (mean ± SD, *n* = 3). Statistical significance was determined by a one-way ANOVA in panels **E** and **G**. *P* value was <0.05(*), <0.01(**), <0.001(***), and <0.0001(****), “ns” indicates no significant difference.

Given that LYN directly interacts with NP and the kinase activity of LYN is the key to restrict IAV replication, we hypothesized that LYN directly catalyzes the phosphorylation of NP. We found that LYN had no significant impact on the serine or threonine phosphorylation of NP but specifically increased the tyrosine phosphorylation of NP (Fig. 4A). In PR8 virus-infected cells, GFP-LYN upregulated the tyrosine phosphorylation of NP, whereas LYN deficiency impaired the tyrosine phosphorylation of NP (Fig. 4B and C). The kinase activity assay showed that NP promotes the ATP consumption of LYN, suggesting NP is a substrate of LYN kinase (Fig. 4D). Moreover, the K275R substitution largely eliminated the tyrosine phosphorylation of NP catalyzed by LYN, and the Y397F/C468A substitutions only partially reduced the tyrosine phosphorylation of NP (Fig. 4E). The *in vitro* kinase assay indicated that LYN directly catalyzes the tyrosine phosphorylation of NP, whereas LYN-K275R lost the ability to catalyze the tyrosine phosphorylation of NP (Fig. 4F). Furthermore, MLR-1023, as an agonist of LYN, promoted the phosphorylation of NP, whereas LYN inhibitors (Bafetinib or LYN-IN-1) blocked the phosphorylation of NP (Fig. 4G).

To map the residues of NP that are specifically modified by LYN, all 13 tyrosine residues within NP were mutated to phenylalanine (Ymuts), and then each residue was mutated back to tyrosine individually (Fig. 4H). Only the 10Y, 40Y, 78Y, and 97Y mutants were tyrosine phosphorylated by LYN, which suggested that residues 10, 40, 78, and 97 of NP are the phosphorylation sites of LYN (Fig. 4H). The quart-site mutation (Y10/40/78/97F) and triple-site mutations of NP were then constructed. NP-Y10/40/78/97F and NP-Y10/40/97F were not phosphorylated by LYN, like the Ymuts with all 13 tyrosine residues mutated to phenylalanine. In contrast, NP-Y10/40/78F, NP-Y10/78/97F, and NP-Y40/78/97F were tyrosine phosphorylated by LYN, indicating that Y10, Y40, and Y97 might be the key residues of NP phosphorylated by LYN (Fig. 4I). An *in vitro* kinase assay confirmed that LYN catalyzes the tyrosine phosphorylation of NP but not NP-Y10/40/97F (Fig. 4J). Alignment of 76,616 NP sequences of all IAVs from GenBank showed that the Y10, Y40, and Y97 residues of NP are highly conserved among IAVs (Fig. 4K). These results indicate that Y10/40/97 are the key sites of NP for direct and specific phosphorylation by LYN.

### Y10/40/97 phosphorylation of NP affects vRNP assembly

**Fig 5:**
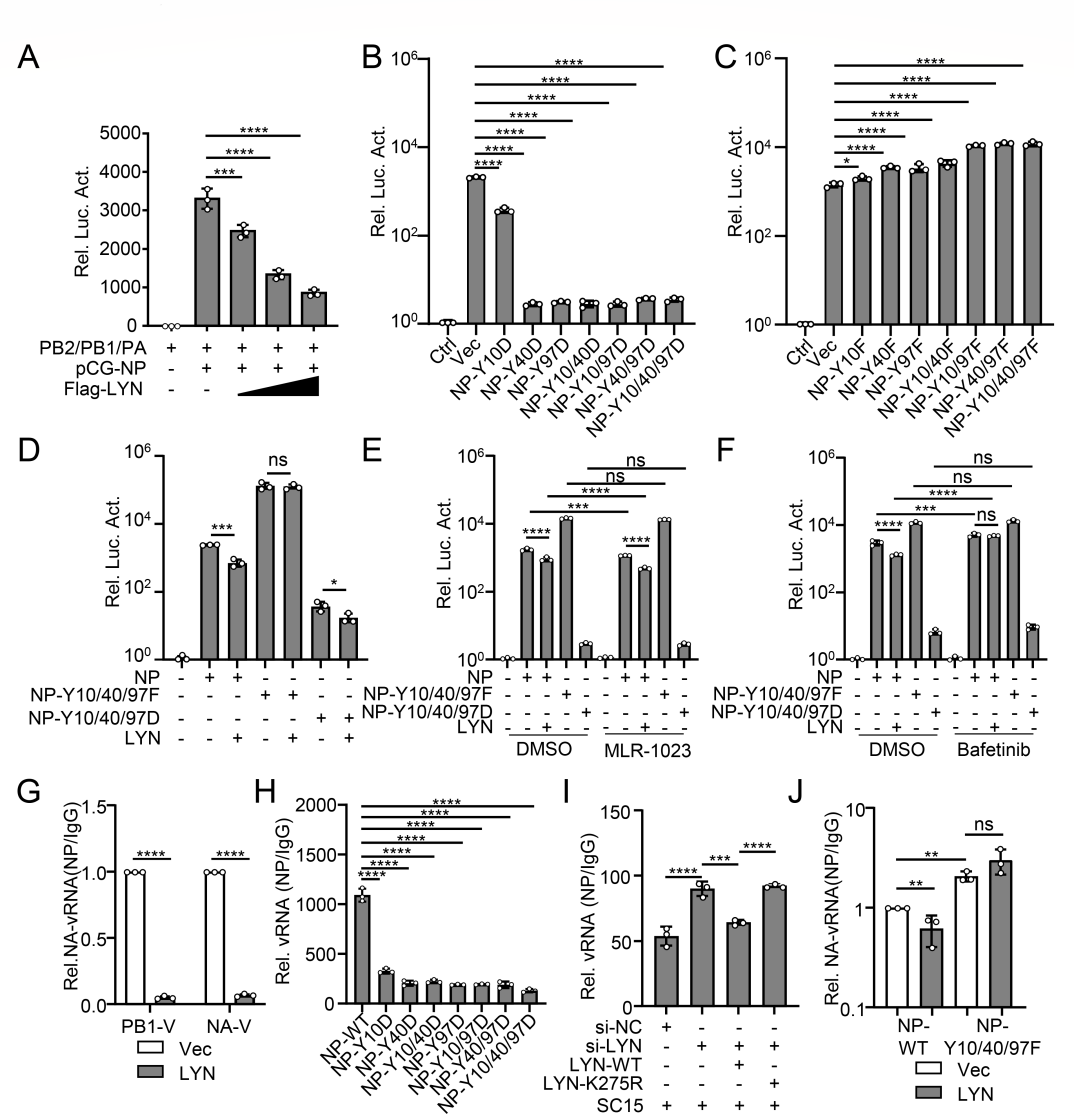
The tyrosine phosphorylation of NP catalyzed by LYN at Y10/40/97 inhibits the viral polymerase activity and the binding of NP with viral RNA. (**A–C**) HEK293T cells were transfected with the indicated plasmids for 24 h. Data shown represent the ratio of luciferase activity normalized to the group without NP (Ctrl). (**D**) HEK293T cells were transfected with the indicated plasmids for 24 h. Data shown represent the ratio of luciferase activity normalized to the group without NP (Ctrl). (**E and F**) HEK293T cells were transfected with the indicated plasmids and treated with MLR-1023 (20 μM) or Bafetinib (20 μM) for 24 h. Data shown represent the ratio of luciferase activity normalized to the group without NP (Ctrl). (**G**) A549 cells were transfected with LYN or Vec for 24 h. The cells were then infected with SC15 virus (MOI = 1) for 24 h before being subjected to the cross-linking immunoprecipitation (CLIP) assay using anti-NP antibody or IgG. The level of PB1 and NA vRNA was quantified by qPCR and normalized to IgG. (**H**) HEK293T cells were transfected with NP or NP mutants, along with PB2, PB1, PA, and pPol I-NA-vRNA vector. After 24 h, the cells were subjected to the CLIP assay with NP-specific antibody or IgG. The level of NA vRNA was quantified by qPCR, normalized to IgG. (**I**) LYN-knockdown (si-LYN) HEK293T cells were transfected with wild-type or mutant LYN for 24 h before being infected with SC15 virus. At 24 h post-infection, the cells were subjected to the CLIP assay with NP-specific antibody or IgG. The level of NA vRNA was quantified by qPCR, normalized to IgG. (**J**) HEK293T cells were transfected with LYN or Vec, along with PB2, PB1, PA, pPol I-NA-vRNA, and NP or NP-Y10/40/97F mutant vector. After 24 h, the cells were subjected to the CLIP assay with NP-specific antibody or IgG. The level of NA vRNA was quantified by qPCR, normalized to IgG. The data represent three independent experiments (mean ± SD, *n* = 3). Statistical significance was determined by a one-way ANOVA in panels **A–D** and **H–I**, or two-way ANOVA in panels** D–G** and **J**. *P* value was <0.05(*), <0.01(**), <0.001(***), and <0.0001(****), “ns” indicates no significant difference.

Since NP is a core component of the vRNP complex along with PB2, PB1, and PA, to initiate viral transcription and replication (27), we tested whether LYN and the phosphorylation of NP (Y10/40/97) affect the polymerase activity in the context of the vRNP complex. The minigenome assay showed that LYN inhibits the polymerase activity in a dose-dependent manner (Fig. 5A). Single, double, and triple Y-D mutation (sustained phosphorylation) of NP at Y10, Y40, and/or Y97 blocked the polymerase activity compared to that with wild-type NP (Fig. 5B). However, single, double, and triple Y-F mutation (dephosphorylation) of NP at Y10, Y40, and/or Y97 increased the polymerase activation of the vRNP complex (Fig. 5C). LYN and MLR-1023 decreased the NP-mediated polymerase activity, and LYN inhibitors increased the NP-mediated polymerase activity, but the treatments with LYN, MLR-1023, or LYN inhibitors did not affect the NP-Y10/40/97F- or NP-Y10/40/97D-mediated polymerase activity (Fig. 5D through F).

NP encapsulates viral RNA to assemble the vRNP complex, in combination with three viral polymerase proteins, and NP oligomerization is essential for maintaining the structure of the vRNP complex (28). The cross-linking immunoprecipitation (CLIP) assay indicated that LYN overexpression and sustained phosphorylation of NP at Y10, Y40, and/or Y97 inhibit the interaction of NP with viral RNAs in virus-infected cells (Fig. 5G and H). LYN knockdown promoted the interaction of NP with viral RNAs, whereas wild-type LYN, but not the LYN-K275R mutant, decreased the interaction of NP with viral RNAs (Fig. 5I). The plasmid pPol I-NA-vRNA was constructed for the expression of NA vRNA under the control of the human RNA polymerase I promoter (29). In cells transfected with PB2, PB1, PA, pPol I-NA-vRNA, and NP expression plasmids, the Y10/40/97F substitution significantly increased the interaction of NP with vRNA derived from plasmids, and overexpression of LYN significantly decreased the interaction of vRNA with wild-type NP, but not NP-Y10/40/97F (Fig. 5J).

**Fig 6:**
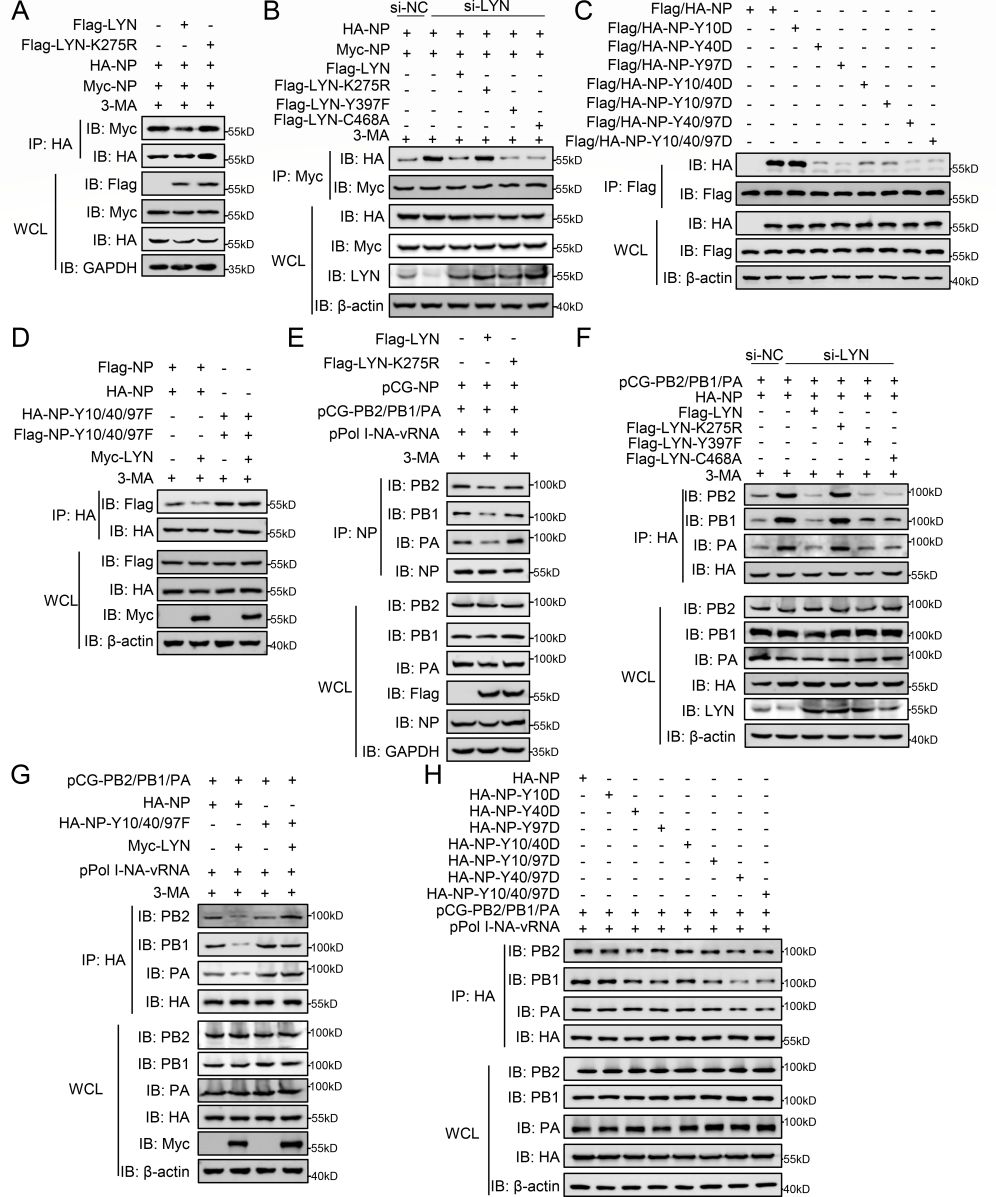
The tyrosine phosphorylation of NP catalyzed by LYN at Y10/40/97 inhibits the assembly of the vRNP complex. (**A**) HEK293T cells were transfected with indicated plasmids for 24 h. The cells were then treated with 3-MA for 6 h before being co-IPed. (**B**) Wild-type (si-NC) or LYN-knockdown (si-LYN) HEK293T cells were transfected with the indicated plasmids for 24 h. The cells were then treated with 3-MA for 6 h before being co-IPed with anti-Myc antibody. (**C–E**) HEK293T cells were transfected with indicated plasmids for 24 h. The cells were then treated with 3-MA for 6 h before being co-IPed with indicated antibodies. (**F**) Wild-type (si-NC) or LYN-knockdown (si-LYN) HEK293T cells were transfected with the indicated plasmids for 24 h. The cells were then treated with 3-MA for 6 h before being co-IPed with anti-HA antibody. (**G and H**) HEK293T cells were transfected with indicated plasmids for 24 h. The cells were then treated with 3-MA for 6 h before being co-IPed with anti-HA antibody. The data shown represent three independent experiments.

Co-IP experiments revealed that LYN, but not LYN-K275R, attenuates the oligomerization of NP (Fig. 6A; Fig. S4A). Knockdown of LYN enhanced the oligomerization of NP, whereas wild-type LYN, LYN-Y397F, and LYN-C468A, but not the LYN-K275R, restored NP oligomerization (Fig. 6B). The multiple sustained phosphorylation mutations of NP inhibited the oligomerization of NP, except for the Y10D substitution (Fig. 6C; Fig. S4B). LYN significantly decreased the oligomerization of wild-type NP, but not NP-Y10/40/97F (Fig. 6D; Fig. S4C).

For the assembly of vRNP, LYN, but not LYN (K275R), impaired the interaction between NP and viral polymerase proteins, which indicates that LYN decreases vRNP assembly (Fig. 6E; Fig. S4D). Knockdown of LYN promoted the assembly of vRNP, whereas wild-type LYN, LYN-Y397F, and LYN-C468A, but not the LYN-K275R, inhibited the assembly of vRNP (Fig. 6F). Furthermore, LYN significantly decreased the interaction of wild-type NP with the viral polymerase proteins, but not NP-Y10/40/97F (Fig. 6G; Fig. S4E). In addition, the multiple sustained phosphorylation mutations of NP inhibited the interaction of NP with PB2/PB1/PA at different levels, and the Y40/97 sites were more important among the three sites (Fig. 6H; Fig. S4F). These results substantiate that LYN-catalyzed phosphorylation of NP at the Y10/40/97 sites is harmful for the assembly and polymerase function of vRNP.

### The NP^Y10/40/97F^ mutation facilitates vRNP assembly and IAV replication in cells

**Fig 7:**
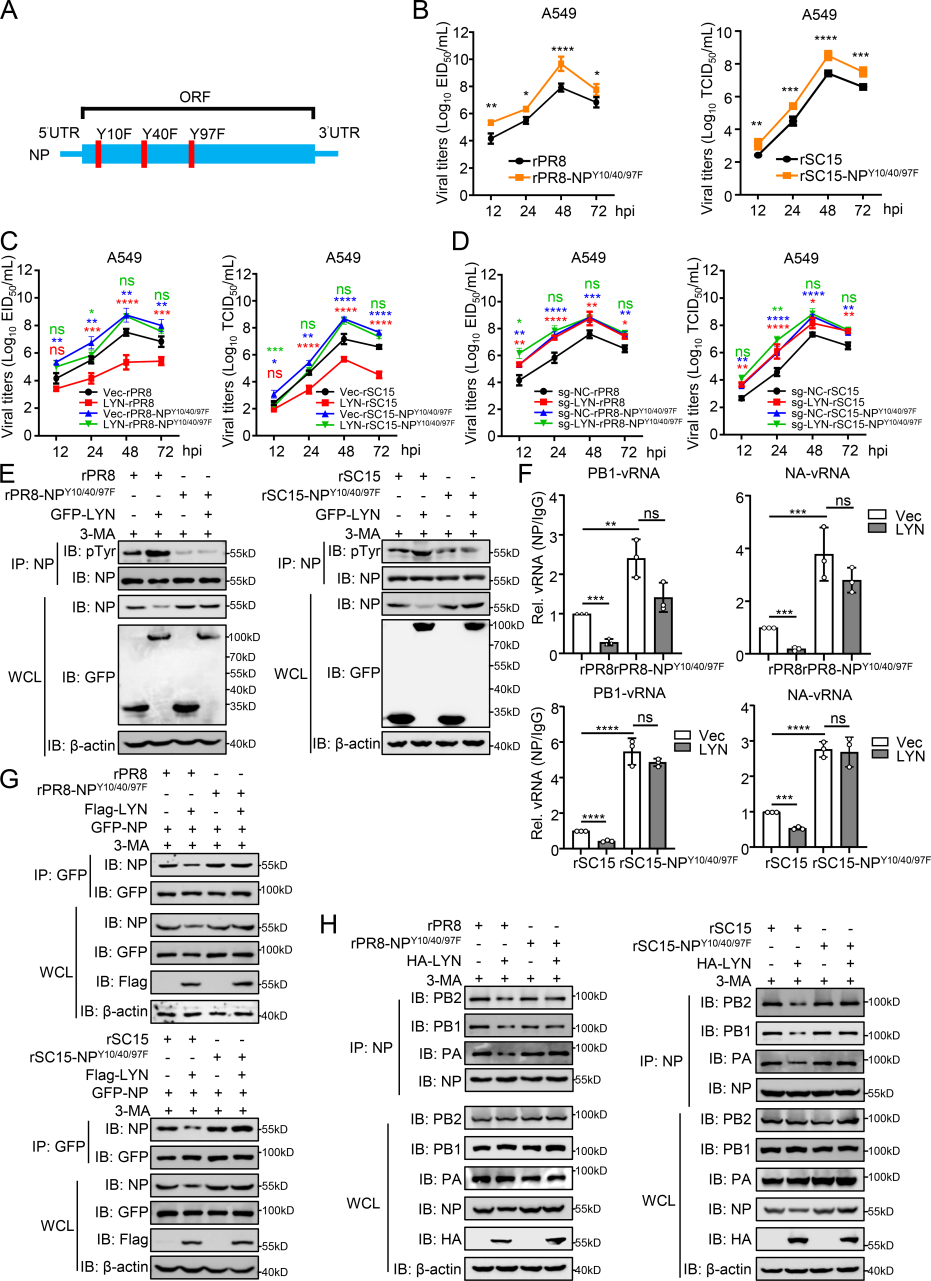
The Y10/40/97F mutation in NP promotes virus replication in cells. (**A**) Schematic illustration of the strategy used to generate NP^Y10/40/97F^ mutant virus. (**B**) A549 cells were infected with rPR8, rPR8-NP^Y10/40/97F^, rSC15, or rSC15-NP^Y10/40/97F^ virus (MOI = 0.01). Supernatants were collected at indicated times post-infection for the EID_50_ or TCID_50_ assay. (**C**) A549 cells were transfected with LYN or Vec for 24 h. Then, the cells were infected with rPR8, rPR8-NP^Y10/40/97F^, rSC15, or rSC15-NP^Y10/40/97F^ virus (MOI = 0.01). Supernatants were collected at different times post-infection for the EID_50_ or TCID_50_ assay. The significant difference between Vec-rPR8/rSC15 and LYN-rPR8/rSC15 is labeled in red; the significant difference between Vec-rPR8/rSC15 and Vec-rPR8/rSC15-NP^Y10/40/97F^ is labeled in blue; the significant difference between Vec-rPR8/rSC15-NP^Y10/40/97F^ and LYN-rPR8/rSC15-NP^Y10/40/97F^ is labeled in green. (**D**) LYN-deficient (sg-LYN) or control (sg-Ctrl) A549 cells were infected with rPR8, rPR8-NP^Y10/40/97F^, rSC15, or rSC15-NP^Y10/40/97F^ virus (MOI = 0.01). Supernatants were collected at the different times post-infection for the EID_50_ or TCID_50_ assay. The significant difference between sg-NC-rPR8/rSC15 and sg-LYN-rPR8/rSC15 is labeled in red; the significant difference between sg-NC-rPR8/rSC15 and sg-NC-rPR8/rSC15-NP^Y10/40/97F^ is labeled in blue; the significant difference between sg-NC-rPR8/rSC15-NP^Y10/40/97F^ and sg-LYN-rPR8/rSC15-NP^Y10/40/97F^ is labeled in green. (**E**) HEK293T cells were transfected with GFP-LYN for 24 h. Then, the cells were infected with rPR8 (MOI = 1), rSC15 (MOI = 1), rPR8-NP^Y10/40/97F^ (MOI = 0.1), or rSC15-NP^Y10/40/97F^ virus (MOI = 0.1); cell lysates were then treated with 3-MA (10 mM) for 6 h before subjected to co-IP and immunoblotting analysis. (**F**) A549 cells were transfected with LYN or Vec for 24 h. The cells were then infected with rPR8 (MOI = 1), rSC15 (MOI = 1), rPR8-NP^Y10/40/97F^ (MOI = 0.1), or rSC15-NP^Y10/40/97F^ virus (MOI = 0.1) for 24 h before being subjected to the CLIP assay using anti-NP antibody or IgG. The level of PB1 and NA vRNA was quantified by qPCR and normalized to IgG. (**G**) HEK293T cells were transfected with LYN or Vec, along with GFP-NP, for 24 h. The cells were then infected with rPR8 (MOI = 1), rSC15 (MOI = 1), rPR8-NP^Y10/40/97F^ (MOI = 0.1), or rSC15-NP^Y10/40/97F^ virus (MOI = 0.1) for 24 h and treated with 3-MA (10 mM) for 6 h before subjected to co-IP and immunoblotting analysis. (**H**) HEK293T cells were transfected with LYN or Vec for 24 h. The cells were then infected with rPR8 (MOI = 1), rSC15 (MOI = 1), rPR8-NP^Y10/40/97F^ (MOI = 0.1), or rSC15-NP^Y10/40/97F^ virus (MOI = 0.1) for 24 h and treated with 3-MA (10 mM) for 6 h before subjected to co-IP and immunoblotting analysis. Statistical significance was determined by a two-way ANOVA in panels B–D and F. The data represent three independent experiments (mean ± SD, *n* = 3). *P* value was <0.05(*), <0.01(**), <0.001(***), and <0.0001(****), “ns” indicates no significant difference.

To determine the significance of NP Y10/40/97 to the IAV replication, we rescued rSC15-NP^Y10/40/97F^ and rPR8-NP^Y10/40/97F^ mutant viruses (Fig. 7A). The viral titration results showed that the replication of rPR8-NP^Y10/40/97F^ and rSC15-NP^Y10/40/97F^ viruses was promoted compared to that of wild-type viruses in cells (Fig. 7B). Overexpression and deficiency of LYN had little effect on the replication of the rSC15-NP^Y10/40/97F^ or rPR8-NP^Y10/40/97F^ mutant viruses (Fig. 7C and D). Co-IP experiments indicated that LYN catalyzed the phosphorylation of NP from rSC15/rPR8 viruses, but not the NP from rSC15-NP^Y10/40/97F^ or rPR8-NP^Y10/40/97F^ mutant viruses (Fig. 7E). Compared with rPR8 or rSC15 virus, the interaction of viral RNAs with NP from rPR8-NP^Y10/40/97F^ or rSC15-NP^Y10/40/97F^ virus was enhanced and not altered by LYN, respectively (Fig. 7F). In addition, in cells infected with rSC15-NP^Y10/40/97F^ or rPR8-NP^Y10/40/97F^ viruses, the oligomerization of NP and the interaction of NP with polymerase proteins were not impaired by LYN, like the wild-type viruses (Fig. 7G and H; Fig. S4G and H). Taken together, these results confirm that the tyrosine phosphorylation of NP at Y10/40/97 is vital for vRNP assembly and virus replication.

### The Y10/40/97F mutation in NP promotes viral replication and pathogenicity *in vivo*

**Fig 8:**
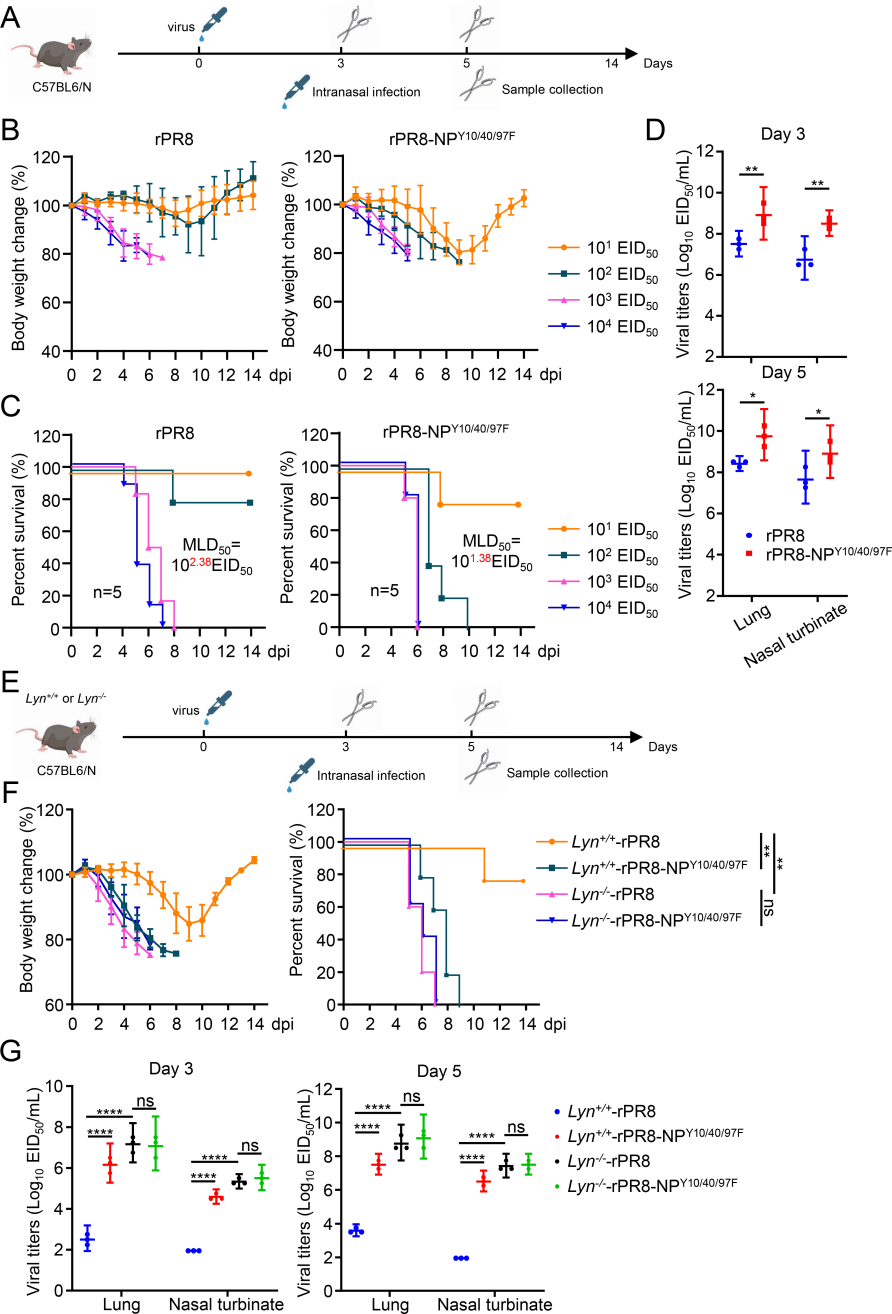
The Y10/40/97F mutation of NP increases the replication and pathogenicity of the H1N1 virus *in vivo*. (**A**) Schematic diagram of rPR8 or rPR8-NP^Y10/40/97F^-infected mice. (**B and C**) Groups of five C57BL/6N mice were intranasally inoculated with 10-fold serial dilutions containing 10^1^ to 10^4^ EID_50_ of rPR8 or rPR8-NP^Y10/40/97F^ virus. Changes in body weight (**B**) and survival (**C**) were monitored for 14 days after virus challenge. The MLD_50_ was calculated by using the method of Reed and Muench. (**D**) Groups of six C57BL/6N mice intranasally infected with 10^4^ EID_50_ of rPR8 or rPR8-NP^Y10/40/97F^ virus were euthanized on days 3 and 5 post-inoculation, and the lungs and nasal turbinate were collected for the EID_50_ assay. Statistical significance was determined by a two-way ANOVA. (**E and F**) Diagram of experimental procedures in mice. Groups of five (*n* = 5) wild-type C57BL/6N (*Lyn^+/+^*) mice and *Lyn^−/−^* mice were intranasally inoculated with rPR8 or rPR8-NP^Y10/40/97F^ viruses. Changes in body weight (left) and survival (right) were monitored daily for 14 days after virus challenge. (**G**) Groups of six C57BL/6N *Lyn^+/+^* or *Lyn^−/−^* mice intranasally infected with 10^2^ EID_50_ of rPR8 or rPR8-NP^Y10/40/97F^ viruses were euthanized on days 3 and 5 post-inoculation, and the lungs and nasal turbinate were collected for the EID_50_ assay. Statistical significance was determined by a two-way ANOVA in panels **D** and **G**. *P* value was <0.05(*), <0.01(**), and <0.0001(****), “ns” indicates no significant difference.

To test the effect of the NP-Y10/40/97F mutation on the virulence of IAVs *in vivo*, we assessed the replication and pathogenicity of the rPR8, rPR8-NP^Y10/40/97F^, rSC15, and rSC15-NP^Y10/40/97F^ viruses in mice (Fig. 8A). Groups of five C57BL6/N mice were intranasally inoculated with serially diluted viruses, and body weight, disease signs, and death were monitored daily for 14 days. Body weight loss of mice inoculated with rPR8 or rSC15 was much less than that of mice inoculated with the same dose of the mutant viruses. As a result, the MLD_50_ of the mutant viruses (10^1.38^ EID_50_ of rPR8-NP^Y10/40/97F^ and 10^1.5^ EID_50_ of rSC15-NP^Y10/40/97F^) was lower than that of the wild-type viruses (10^2.38^ EID_50_ of rPR8 and 10^2.38^ EID_50_ of rSC15, Fig. 8B and C; Fig. S5A and B). The viral loads of the nasal turbinate and lungs from mice infected with the rPR8-NP^Y10/40/97F^ and rSC15-NP^Y10/40/97F^ mutant viruses were significantly higher than those from mice infected with the wild-type viruses (Fig. 8D; Fig. S5C). In addition, more NP-specific signals in the lungs of mice infected with the mutant viruses were detected when compared to those infected with wild-type viruses at day 3 post-infection (Fig. S5D, F, S6A and C). Consistent with these data, the pathological findings indicated that infection with mutant viruses led to recruitment of more lymphocytes and neutrophils to the lungs and induced more severe lesions (Fig. S5E, G, S6B and D).

To further confirm the importance of LYN-catalyzed phosphorylation of NP at the Y10/40/97 sites to the IAV replication, groups of five *Lyn^+/+^* and *Lyn^–/–^* mice were intranasally inoculated with the rPR8 or rPR8-NP^Y10/40/97F^ viruses (Fig. 8E). As shown in Fig. 8F, both the NP-Y10/40/97 mutation and the LYN deficiency increased the infected-induced weight loss and mortality, but the wild-type rPR8 and rPR8-NP^Y10/40/97F^ mutant viruses exhibited no significant difference for the weight loss and mortality in *Lyn^–/–^* mice. Consistently, the viral loads of the nasal turbinate and lung tissues from the rPR8-NP^Y10/40/97F^-infected *Lyn^+/+^* mice and the rPR8-infected *Lyn^–/–^* mice are much higher than those from the rPR8-infected *Lyn^+/+^* mice, while viral loads of tissues from rPR8- or rPR8-NP^Y10/40/97F^-infected *Lyn^–/–^* mice showed similar levels (Fig. 8G). These results collectively demonstrate that the LYN-catalyzed phosphorylation of NP at the Y10/40/97 sites is critical for the replication and pathogenicity of IAV in mice.

## DISCUSSION

Host factors play a vital role in the life cycle of IAVs. In the present study, we identified a novel IAV-related host factor, LYN, through affinity purification mass spectrometry. Our findings lead us to propose a model in which LYN attenuates IAV replication by regulating the phosphorylation of NP, specifically at the novel functional phosphorylation sites Y10, Y40, and Y97 (Fig. S7). Phosphorylation of NP at Y10, Y40, and Y97 inhibits the RNA binding activity, oligomerization, and interaction with viral polymerase of NP, thereby disrupting vRNP assembly and viral polymerase activity, which are crucial for virus replication. In addition, the LYN agonist MLR-1023 was found to enhance NP phosphorylation and suppress IAV proliferation and virulence. These findings reveal that LYN serves as a novel negative regulator of IAV replication via tyrosine phosphorylation.

Kinases are proteins that participate in almost all cellular signaling processes by phosphorylating other proteins, lipids, or carbohydrates (30). The human kinome comprises 538 kinases (so far) that are categorized into different types, including serine/threonine kinases, receptor tyrosine kinases (RTKs), non-RTKs, histidine kinases, dual specificity kinases, and lipid kinases (31, 32). Few kinases have been reported to be involved in the life cycle of IAV; however, we know that the binding of IAV to cells leads to the clustering of lipid rafts and activation of the RTK family member epidermal growth factor receptor, which in turn facilitates IAV uptake (33). During the release of the IAV genome into the cytoplasm, the kinases ERK and PI3K are activated by IAV infection, which leads to the activation of vacuolar (H^+^)-ATPases, the acidification of endosomes, and fusion between the viral and endosomal membranes (34). Recently, two Ser/Thr protein kinases, ATM and CK2, have been related to the phosphorylation of S23, S24, and S25 (S-S-S motif) of NEP and further regulate the vRNP nuclear export and the virulence of IAV (35). Another serine/threonine kinase, PLK3, was reported to interact with NP, significantly increase NP phosphorylation and oligomerization, and to promote viral ribonucleoprotein assembly and replication (36). Nevertheless, these kinases are all serine/threonine kinases, but not tyrosine kinases. And no direct evidence, like an *in vitro* kinase assay, was supplied to prove the direct catalyzing of kinase to the viral proteins. In the present study, we revealed that a novel tyrosine kinase, LYN, restricts the replication of various IAV *in vitro*, and *Lyn^–/–^* mice are more sensitive to the infection of IAV (Fig. 1 and 2). In addition, LYN directly interacts with NP and catalyzes the tyrosine phosphorylation of NP (Fig. 3 and 4).

LYN, as a member of the SRC family, has been reported to be involved in cell proliferation, migration, metabolism, tumorigenesis, and especially the inflammation (37). LYN also plays a pivotal role in the regulation of allergic reactions through interactions with immunoglobulin E and its high-affinity receptor (FcεRI) (38). Moreover, LYN has been found to be involved in viral infections in which viral proteins modulate the kinase activity of LYN to influence host cell processes, like proliferation, apoptosis, and immune activity. For example, flaviviruses exploit Lyn-dependent transport in autophagosomes to evade circulating antibodies (24). Human immunodeficiency virus type 1 or simian immunodeficiency virus infection leads to the accumulation of inflammatory monocytes, and viral protein Nef is responsible for these phenotypes via a LYN-dependent mechanism (39). LYN has also been reported to be expressed in B and myeloid immune cells, regulating innate and adaptive immunity, such as Toll-like receptor signaling and the B cell receptor complex signaling, which might be partially responsible for the anti-influenza effect of LYN (40–42). However, it was unclear whether direct regulation of LYN could affect the function of viral proteins and viral replication through the kinase activity of LYN. As shown in Fig. 3 and Fig. S3, the K275R mutation of LYN, lost the kinase activity and reduced the inhibitory effect of LYN on IAV replication. Furthermore, treatment with LYN agonist MLR-1023 inhibited IAV replication, and treatment with LYN inhibitors blocked the inhibitory effect of LYN on IAV replication. More importantly, LYN directly phosphorylates NP at tyrosine 10/40/97 (Fig. 4). The present study demonstrates that kinase LYN acts as an anti-influenza factor by directly catalyzing the phosphorylation of the viral protein NP and reducing the replication of the virus.

NP is the most abundant component of the IAV vRNP complex, and its functions, such as encapsulation of viral RNA, oligomerization, and assembly of the RNP, are essential for successful infection. Post-translational modifications, such as phosphorylation, play a crucial role in the stability and functionality of NP. Specific phosphorylation sites on NP, such as S165, S407, and S486, impact oligomerization and the polymerase activity of the vRNP complex (8, 12). Phosphorylation at S9, Y10, T188, and Y296 regulates NP nuclear-cytoplasmic shuttling and polymerase activity, whereas Y78 phosphorylation delays nuclear export (11, 43, 44). Y385 phosphorylation of NP confers temperature sensitivity to influenza A virus due to impaired NP oligomerization at a lower temperature (45). Phosphomimic mutants (e.g., S165E, S165D, S407E, S413E) block homotypic interactions and drive NP toward a monomeric form (8, 12, 46). In the present study, the Y10, Y40, and Y97 residues were identified as the phosphorylation sites of NP catalyzed by LYN, as confirmed by *in vitro* assays (Fig. 4). LYN-mediated phosphorylation or mimicking phosphorylation at these residues inhibits NP self-oligomerization and weakens the interactions of NP with polymerase proteins and viral RNA, resulting in reduced viral polymerase activity and virus replication (Fig. 5 to 8). In short, phosphorylation is a key regulatory mechanism of NP function and the life cycle of IAV.

While substantial efforts and progress have been made in the development of anti-influenza drugs, influenza still poses a severe public health burden due to drug resistance. The existing drugs for IAV are designed to target viral proteins, which frequently exhibit resistance to these drugs when new virus strains emerge through acquired mutations in the viral genome (47). Therefore, host-directed therapy could lead to novel, longer-lasting, and effective therapeutics and antiviral strategies. Given that phosphorylation events are essential for biomolecular structural transformation, functional activation, molecular interaction, and subcellular localization, kinases might be promising antiviral targets (48). Small molecular compounds targeting kinases could impede the activity of specific signaling pathways to regulate enzymes that are involved in the growth and proliferation of cells and viruses. Kinase inhibitors and agonists may prove a superior alternative to traditional antiviral drugs, given the inherent difficulty for the virus to circumvent the host dependency. The present study demonstrated that LYN kinase, encoded by the *LYN* gene, is a promising drug target for IAV infection. Moreover, the LYN agonist MLR-1023 showed robust efficacy against IAV in cells and effectively reduced the viral load of IAV in mice (Fig. 3). Since March 2024, highly pathogenic H5N1 avian influenza viruses have been transmitted across interspecies barriers to mammals such as dairy cows and even humans in the United States (4, 49). In this study, we found that LYN displayed efficient antiviral effects against different subtypes of influenza viruses, including H5 IAV, and that LYN significantly inhibited virus replication in cells derived from different species, including MDBK from bovine. Therefore, the LYN agonist MLR-1023 may be an alternative drug candidate for emergency medications for the H5N1 virus in the United States. Despite the advantages, kinase inhibitors and agonists are subject to off-target effects and associated toxicity, which must be minimized to enhance the efficacy as drugs (32). The discovery of LYN as a potential drug target opens new avenues for the development of host-directed antiviral therapies. Future investigation could focus on optimizing LYN agonists, exploring their efficacy against other influenza strains, and evaluating their safety and efficacy in clinical trials.

In summary, our study found that the host kinase LYN plays a crucial role in the life cycle of IAV, attenuating IAV replication by catalyzing tyrosine phosphorylation of NP. In addition, we identified the tyrosine phosphorylation sites Y10/40/97 on NP targeted by LYN and found that the phosphorylation of NP at Y10/40/97 inhibits NP self-oligomerization and weakens the interactions of NP with polymerase proteins and viral RNA, resulting in impaired assembly of vRNP and the viral polymerase activity, which in turn attenuates the replication of IAV *in vitro* and *in vivo*. This work broadens our understanding of the host-IAV interaction and provides new ideas for the development of anti-viral drugs. LYN may also become the next target for the treatment of IAV. In-depth illustration of virus-host kinase interplay is essential for a comprehensive understanding of virus-host interactions and may lay the foundation for the application of kinase inhibitors and agonists as novel anti-influenza drugs.

## MATERIALS AND METHODS

### Cells, viruses, and plasmids

MDCK, MDBK, HEK293T, U2OS, BHK-21, RAW264.7, and HD11 cells were grown in DMEM (Gibco) supplemented with 10% (vol/vol) FBS (Gibco-BRL; 10099-141) and 1× penicillin/streptomycin (Gibco-BRL; 10378016). A549 cells were grown in Kaighn's modified Ham F-12 nutrient mixture medium (Gibco) supplemented with 10% FBS and penicillin/streptomycin. All cells were cultured and maintained at 37°C with 5% CO_2_. All cells were purchased from the American Type Culture Collection (ATCC, USA) and were tested for mycoplasma contamination.

The H1N1 influenza A virus (A/Puerto Rico/8/1934, PR8) was stored in our laboratory. The H5N6 IAV (A/Goose/Sichuan/SC15/2015, SC15) was isolated in Guangdong Province in China in 2015 (29). The H7N9 virus (A/Suzhou/SZ19/2014, SZ19) was isolated from Jiangsu Province in China in 2014 (50). The H9N2 virus (A/chicken/Hunan/38/2018, HN38) was isolated from Gansu Province in China in 2018. The recombinant SC15 reporter virus expressing Nanoluciferase (SC15-Nluc) was generated as previously described (51). Virus stocks were propagated in specific-pathogen-free (SPF) chicken eggs and stored at −70°C until use.

Human LYN, LYN mutants (K275R, Y397F, C468A), and LYN truncations were constructed by using standard molecular biology techniques. The genes encoding PB1, PB2, PA, and NP were amplified from the PR8 or SC15 viral genomes and then cloned into the pCAGGS (pCG) vector. Single or multiple point mutants of the NP genes (generated by using a PCR approach) were cloned into the pCG vector. The plasmid pPol I-Luc, for the expression of a viral RNA-like firefly luciferase gene under the control of the human RNA polymerase I promoter, has been reported previously (52). The negative strands of the viral NA and NP genes were amplified and cloned into the plasmid pPol I for the expression of vRNA under the control of the human RNA polymerase I promoter (29).

### Reagents and antibodies

The antibodies used in this study were as follows: HRP-conjugated anti-HA (12013819001), anti-Myc (11814150001), and anti-GFP (11814460001) antibody (Roche); rabbit anti-PB2 (GTX125926), anti-PB1 (GTX125923), and anti-PA (GTX118991) polyclonal antibodies (Genetex); Phospho-LYN (Tyr397, 70926S) (Cell Signaling Technology); p-Ser/phosphoserine antibody (sc-81514), p-Thr/phosphothreonine (sc-5267) and p-Tyr/Phosphotyrosine antibody (sc-508) (Santa Cruz); HRP-conjugated anti-Human IgG-Fc (SSA001) and anti-NP (11675-MM03T) antibody (Sino Biological); anti-β actin (TA-09), anti-His tag (TA-02), and HRP-conjugated goat anti-rabbit IgG (ZB-2301) (Zsbio); HRP-conjugated goat anti-mouse secondary antibody (ab102448), anti-LYN (ab137338), and anti-GAPDH (ab181602) antibodies (Abcam); goat anti-Human IgG (Fc specific) antibody (F9512) and HRP-conjugated anti-Flag (A8592) antibody (Sigma); and Cy3-labeled goat anti-rabbit IgG (A0516, Beyotime). The anti-IAV-NP nanobody (C7), fused with human IgG Fc fragment, was selected and produced based on previous descriptions (53).

Reagents used in the study included anti-Flag agarose affinity beads (A2220), protease inhibitor cocktail (4693116001), and protein A/G agarose affinity beads (P6486/E3403) (Merck); DAPI (C1002), NP-40 (ST366), 4% paraformaldehyde fix solution (P0099), RNase A (ST578), normal Rabbit IgG (A7016), polybrene (ST1380), puromycin dihydrochloride (ST551), cell counting kit-8 (CCK8, C0037), and streptavidin magnetic beads (P2151) (Beyotime); protein A/G magnetic beads (88803) (Thermo Fisher Scientific); MLR-1023 (T6896), Bafetinib (T6311), and Lyn-IN-1 (T11916) (TargetMol); phosphatase inhibitor cocktail (HY-K0023), 3-methyladenine (3-MA, HY-19312), dimethyl sulfoxide (DMSO, HY-Y0320), polyethylene glycol 300 (PEG300, HY-Y0873A), baloxavir (HY-109025A), and Tween-80 (HY-Y1891) (MedChemExpress). Transfection reagent jetPRIME was obtained from Polyplus, and RNAi MAX was obtained from Invitrogen (USA). SYBR Green I Master Mix was purchased from Roche (Germany).

### Tandem affinity purification of SFB-tagged protein complexes

The gene encoding NP was subcloned into pDONOR201 vector using Gateway Technology (Invitrogen) as the entry clone. The entry clone was subsequently recombined into a lentiviral-gateway-compatible destination vector for the expression of SFB-tagged NP. HEK293T cells were transfected with SFB-NP construct. Twenty-four hours later, the cells were selected by culturing in medium containing puromycin (2 μg/mL), and protein expression was confirmed by immunostaining and western blotting. For affinity purification, HEK293T cells stably expressing SFB-NP were lysed in NETN lysis buffer (100 mM NaCl, 20 mM Tris-HCl, 0.5 mM EDTA, 0.5% NP-40) with protease and phosphatase inhibitors at 4°C for 20 min. The cell lysates were then centrifuged at 4°C and 14,000 rpm for 15 min. Supernatants were incubated with streptavidin-conjugated beads for 1 h at 4°C. The beads were washed three times with NETN buffer, and bound proteins were eluted with NETN buffer containing 2 mg/mL biotin for 90 min at 4°C. The eluates were incubated with S-protein beads for 1 h at 4°C. The beads were then washed three times with NETN buffer and subjected to mass spectrometry. The mass spectrometry analysis was carried out on a Q-Exactive HF-X platform (Thermo Scientific) at the Shanghai Baipu Biotechnology Co., Ltd., following the vendor’s recommended protocol.

### RNA interference

The siRNA library targeting the 50 selected kinase genes, siRNA targeting LYN, and scrambled siRNA (si-NC) were purchased from RiboBio Co. (China); the sequences of the siRNAs are listed in Table S2. All transfections with siRNA were performed according to the manufacturer's instructions using Lipofectamine RNAiMAX reagent.

The siRNA library screening assay was performed in 24-plates. The siRNA-transfected A549 cells were maintained at 37°C for 72 h before they were infected with SC15-Nluc virus (multiplicity of infection [MOI] = 0.01). The supernatants of the samples were collected for the luciferase assay at the indicated time points, followed by centrifugation at 2,000 × *g* for 5 min at 4°C. The luciferase activity of the samples was measured using a Nano-Glo Luciferase Assay System (Promega, USA) according to the manufacturer′s instructions. Non-targeting scramble siRNA (si-NC)-transfected cells were used as negative controls.

Short hairpin RNA (shRNA) constructs were designed and cloned into the pLKO.1-EGFP-puro lentiviral backbone as per the manufacturer's protocol (Tsingke Biotechnology, China). The target sequences for LYN are listed in Table S3. The sequence of nonsense shRNA was provided by Tsingke. Lentivirus was produced in HEK293T cells transfected with viral constructs along with psPAX2 and pMD2G constructs. Viral supernatants were collected on days 2 and 3 after transfection and used to infect target cells.

### Viral infection and titration

All cells were seeded at the desired density in culture plates as per the requirements for different experiments. Viruses were inoculated into cells at a specific MOI for different experiments. One hour after inoculation, the medium was replaced with fresh OPTI-MEM, and the cells were incubated at 37°C. Virus-containing culture supernatants were collected at the indicated time points for titration. Virus titers of virus stocks, cell culture supernatants, and tissue suspensions were determined by end-point titration in MDCK cells or eggs. For end-point viral titration in MDCK cells, 10-fold serial dilutions of each sample were inoculated into MDCK cells. Two days after inoculation, supernatants from the inoculated cells were collected and tested for the ability to agglutinate chicken erythrocytes as an indicator of viral replication. Infectious virus titers are reported as log_10_ TCID_50_/mL and were calculated from three replicates by the method of Reed–Muench (54). For end-point viral titration in eggs, 10-fold serial dilutions of each sample were inoculated into 9-day-old SPF eggs. Sixty hours after inoculation, fluid from the allantoic cavity was collected and tested for its ability to agglutinate chicken erythrocytes as an indicator of viral replication. Infectious virus titers are reported as log_10_ EID_50_/mL and were calculated from three replicates by using the method of Reed–Muench (55).

### CRISPR-Cas9 knockout

Genome engineering was performed using the CRISPR-Cas9 system (56). Double-stranded oligonucleotides corresponding to the target sequences were cloned into the pX459 vector. Two micrograms of each pX459 plasmid containing one of the targeting sequences were simultaneously transfected into A549 or HEK293T cells. The transfected cells were selected with puromycin for at least 7 days to obtain knockout cell pools. The sequences that were targeted for the human *LYN* gene are listed in Table S4.

### Mouse study

*Lyn^−/−^* mice were provided by Cyagen Biosciences Inc. Mice were genotyped from tail biopsies using the primers listed in Table S5. All mice were on a C57BL/6N background and were maintained under pathogen-free conditions. Mice aged 6–8 weeks were used in this study. To determine MLD_50_ values, groups of five 6-week-old female wild-type (*Lyn^+/+^*) or *Lyn^−/−^* C57BL/6N mice were lightly anesthetized with CO_2_ and inoculated intranasally with 10-fold serial dilutions of wild-type or mutant viruses in a 50-µL volume. The mice were monitored daily for 14 days for weight loss and mortality. To assess virus replication and tissue lesions, groups of three 6-week-old female *Lyn^+/+^* or *Lyn^−/−^* mice were lightly anesthetized with CO_2_ and inoculated intranasally with 10^4^ EID_50_ of wild-type or mutant viruses in a 50-μL volume and euthanized on days 3 and 5 post-infection. The lung and nasal turbinate were collected for viral titration or for immunofluorescence and pathological assessments by Servicebio (Servicebio Technology, China).

To evaluate the effect of the LYN agonist (MLR-1023) and inhibitors (Bafetinib and Lyn-IN-1) on the replication and pathogenicity of IAV *in vivo*, the drugs were dissolved to the desired concentrations in a suspension of vehicle (10% DMSO, 40% PEG300, 5% Tween-80, and 45% saline). Drug treatment was initiated 7 days before the challenge and maintained for 14 days, once daily, through intraperitoneal injections. The mice were randomly distributed into the different groups (11 per group), lightly anesthetized with CO_2_ and inoculated via the intranasal route with 50 μL of an inoculum containing either PBS (mock group) or 10^4^ EID_50_ of SC15 virus. After inoculation, five mice in each group were monitored daily for 14 days for weight loss and mortality. The other six mice per group were euthanized on days 3 and 5 post-infection, and their lung and nasal turbinate tissues were collected for viral titration or for immunofluorescence and pathological assessments by Servicebio (Servicebio Technology, China).

### RNA isolation and qRT-PCR

Total RNA from cells or other samples was extracted with TRIzol, following the manufacturer's instructions. For cellular mRNAs, total RNA was subsequently transcribed into cDNA using M-MLV reverse transcriptase, according to the manufacturer's protocol (Promega). The levels of viral vRNA, cRNA, and mRNA were determined by using a strand-specific real-time RT-PCR as described previously (29). GAPDH, actin, or HPRT1 was used as a control for the normalization of cellular mRNA and intracellular viral RNA. Real-time PCR was carried out using the ABI 7500 Detection System (Applied Biosystems, USA). The RNA level of each gene is shown as a fold of induction (2^−ΔΔCT^) in the graph. The sequences of the primers used for qRT-PCR are shown in Table S5.

### Dual-luciferase reporter assay

HEK293T cells were transfected with pCG constructs expressing viral PB2, PB1, PA, and NP or NP mutants, the construct pPol I-Luc, and an internal control pRL-TK (Promega), along with plasmids encoding the indicated proteins. Cells were incubated at 37°C for 24 h, and cell lysates were subsequently prepared by using the Dual-Luciferase Reporter Assay System (Promega). The luciferase activities were measured on a GloMax 96 microplate luminometer (Promega) as reported previously (52).

### Cross-linking immunoprecipitation assay

The CLIP assay was conducted as described previously (29). Briefly, cells were cross-linked and lysed at the indicated times post-infection or transfection. The lysed cell suspension was centrifuged for 5 min at 10,000 × *g* at 4°C, and the supernatant was incubated with specific antibodies, or IgG for 4 h at 4°C. Then the mixture was incubated with protein A/G magnetic beads and rotated for 4 h at 4°C. After the incubation, the beads were washed three times, followed by incubation with 50 μg of proteinase K (Takara Bio) at 55°C for 30 min. Input and co-immunoprecipitated RNAs were recovered by TRIzol, extraction, and analyzed by qPCR.

### Generation of mutant viruses by reverse genetics

Mutant viruses were generated by using the reverse genetics system as described previously (57). Briefly, the eight gene segments of the SC15 and SC15 mutant viruses were inserted into the vRNA-mRNA bidirectional transcription vector pBD. HEK293T cells at 80%–90% confluence in six-well plates were transfected with 5 μg of the eight plasmids (about 0.6 μg/plasmid). Sixteen hours later, the DNA-transfection reagent mixture was replaced with OPTI-MEM. Forty-eight hours after the transfection, the supernatants were harvested and inoculated into embryonated eggs for virus propagation. Viruses were detected by using a hemagglutination assay and were fully sequenced to ensure the absence of unwanted mutations.

### Western blotting

Cells or protein samples were lysed in RIPA buffer (Beyotime, China). Proteins were separated by 10% SDS-PAGE and transferred to a nitrocellulose membrane (Bio-Rad). The membrane was blocked for 1 h in TBST containing 5% milk and subsequently incubated with primary antibodies overnight at 4°C. After a 1-h incubation with HRP-conjugated secondary antibody, the immunoreactive bands were visualized using an e-BLOT system (e-BLOT Life Science, China). The intensities of the target bands were quantified by using the Image J program (NIH, USA).

### GST pull-down assay

GST pull-down assays were conducted as previously described (50). Briefly, the encoded GST- or His-tagged fusion proteins and the control GST proteins were expressed in BL21 cells and purified through Glutathione–Sepharose 4B beads or Ni-BestaRose FF, in accordance with the manufacturer's instructions. Then, 1 μg of purified GST protein or GST fusion protein was captured by the Glutathione–Sepharose 4B beads (GE Healthcare), and purified His-tagged proteins or cell lysates containing the indicated protein were added for incubation overnight at 4°C. The beads were then washed three times with ice-cold PBS. The supernatant was loaded onto gels, followed by immunoblotting analysis.

### Co-immunoprecipitation

HEK293T cells or A549 cells were co-transfected with the indicated plasmids with or without virus infection for 24 h. The transfected cells were then harvested and lysed in NP-40 lysis buffer (20 mM Tris-HCl [pH 7.5], 150 mM NaCl, 1% NP-40, 1 mM EDTA with protease inhibitor cocktails). For each immunoprecipitation, 1 mL of lysate or lysate mixture was incubated for 4 h at 4°C with 0.5 μg of the indicated antibody or control IgG and 30 μL of protein A/G-Sepharose (Sigma). The beads were washed three times with 1 mL of lysis buffer containing 500 mM NaCl. The precipitates were then analyzed by using standard immunoblotting procedures.

### Confocal microscopy

Confocal microscopy was performed as previously described (50). Cells were seeded in 12-well plates on coverslips. At 24 h after transfection, the cells were left uninfected or were infected with IAV for the indicated times before fixing with 4% paraformaldehyde. Cells were washed three times with PBS, permeabilized with 0.1% Triton X-100 in PBS for 10 min, and blocked with 5% skimmed milk for 1 h. Then, the cells were incubated with the indicated primary and secondary antibodies and DAPI. The stained cells were observed with a Leica microscope (TCS SP8) with a 100× oil objective, NA 1.40.

### *In vitro* phosphorylation assay

The *in vitro* phosphorylation assay was performed as previously described (58). For GST- or His-tagged LYN, LYN mutants*,* or NP, the plasmids were transduced into *Escherichia coli* BL21 competent cells for expression and purification. For Flag- or HA-tagged LYN, LYN mutants*,* NP, or other proteins, the plasmids were individually transfected into HEK293T cells, and the proteins were then purified with Protein G Sepharose beads. Then, the indicated purified proteins were co-incubated in an equal volume of 2× reaction buffer (100 mM Tris-HCl, 20 mM MgCl_2_, 1 mM Na_3_VO_4_, 4 mM DTT, pH 7.2), and 1 mM ATP was added prior to incubation for 1 h at 30°C. The reaction was stopped by adding 1/5 volume of 6× SDS loading buffer and boiling the samples for 15 min at 95°C before immunoblot analysis.

### LYN kinase assay

The assay for LYN kinase activity was carried out using the Kinase-Lumi Plus Luminescent Kinase Assay Kit (Beyotime, China). Purified LYN or LYN-K275R (final concentration 30 nM), with or without the mixture of NP (final concentration 10 nM), was prepared in assay buffer (100 mM Tris-HCl, 20 mM KCl, and 6 mM MgCl_2_, pH 7.4). The reaction was started by adding ATP (final concentration 10 μM) at 25°C. The visualization reagents from the Luminescent Kinase Assay kit were added for 20 min. The amplified chemiluminescence signal was determined using a GloMax 96 microplate luminometer (Promega). The amount of luminescence from each reaction is inversely correlated with kinase activity.

### Statistical analyses

Data are expressed as the mean ± standard deviation (SD). Statistical significance was determined by using the Student's two-tailed unpaired *t*-test or analysis of variance (ANOVA) with GraphPad Prism software (version 8.0, San Diego, CA, USA). Differences between groups were considered significant when the *P* value was <0.05(*), <0.01(**), <0.001(***), and <0.0001(****), “ns” indicates no significant difference.

## Data Availability

All data are included in the article and/or Supplementary Data.

## SUPPLEMENTAL MATERIAL

Supplemental figures mbio.03759-25-s0001.pdf Figures S1 to S7.

Supplemental tables mbio.03759-25-s0002.xlsx Tables S1 to S5.

ASM does not own the copyrights to Supplemental Material that may be linked to, or accessed through, an article. The authors have granted ASM a non-exclusive, world-wide license to publish the Supplemental Material files. Please contact the corresponding author directly for reuse.
