# Supplementary material for: Tyrosine kinase LYN restricts the replication and virulence of influenza A virus by directly phosphorylating viral nucleoprotein
Source: mBio. 2026 Mar 30;17(5):e03759-25. doi: 10.1128/mbio.03759-25 (PMC13170340; doi:10.1128/mbio.03759-25)
Supplement: Supplemental figures — Figures S1 to S7. [file mbio.03759-25-s0001.pdf]

Supplementary figures for

Tyrosine kinase LYN restricts the replication and virulence of influenza A virus by directly phosphorylating viral nucleoprotein

Junwen Liu[a], Jiaxin Huang[a], Qian Wang[a], Yanli Wei[a], Lebin Han[a], Xin Li[a], Caoqi Lei[c], Guohua Deng[b], Hualan Chen[b], Qiyun Zhu[a,d,*] and Shuai Xu[a,d,*]

[a] State Key Laboratory of Animal Disease Control and Prevention, College of Veterinary Medicine, Lanzhou University, Lanzhou Veterinary Research Institute, Chinese Academy of Agricultural Sciences, Lanzhou, 730000, PR China

[b] State Key Laboratory of Animal Disease Control and Prevention, Harbin Veterinary Research Institute, Chinese Academy of Agricultural Sciences, Harbin, 150069, PR China

[c] School of Basic Medical Sciences, Lanzhou University, Lanzhou 730000, PR China.

[d] Gansu Province Research Center for Basic Disciplines of Pathogen Biology, Lanzhou 730046, PR China

[*] To whom correspondence should be addressed. Email: xushuai@caas.cn or zhuqiyun@caas.cn

This file includes:

Figures S1 to S7

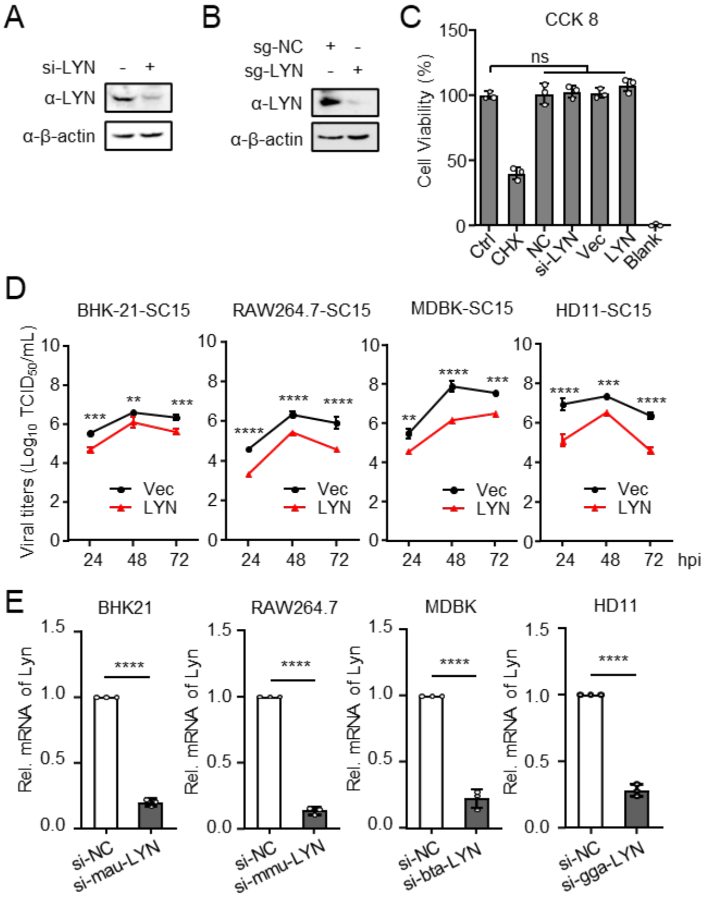

Figure S1. LYN attenuates the replication of IAV *in vitro*. (A) A549 cells were transfected with either the LYN siRNA or NC for 24 h before being analyzed by western blotting. (B) The expression of LYN in LYN-deficient (sg-LYN) or control (sg-Ctrl) A549 cells. (C) Effect of LYN overexpression and knockdown on cell viability detected by CCK8. (D) BHK-21, RAW264.7, MDBK, and HD11 cells were transfected with either the LYN expression plasmid or Vec for 24 h before infection with SC15 virus (MOI = 0.01). Supernatants were analyzed at the indicated times post-infection by using the $TCID_{50}$ assay. (E) BHK-21, RAW264.7, MDBK, and HD11 cells were transfected with either si-NC or si-RNA targeting the species-specific LYN gene, respectively. At 24 h post transfection, the cells were collected for qPCR analysis. Statistical significance was determined by a one-way ANOVA in C and E, or two-way ANOVA in D. The data represent three independent experiments (mean ± SD, n = 3).

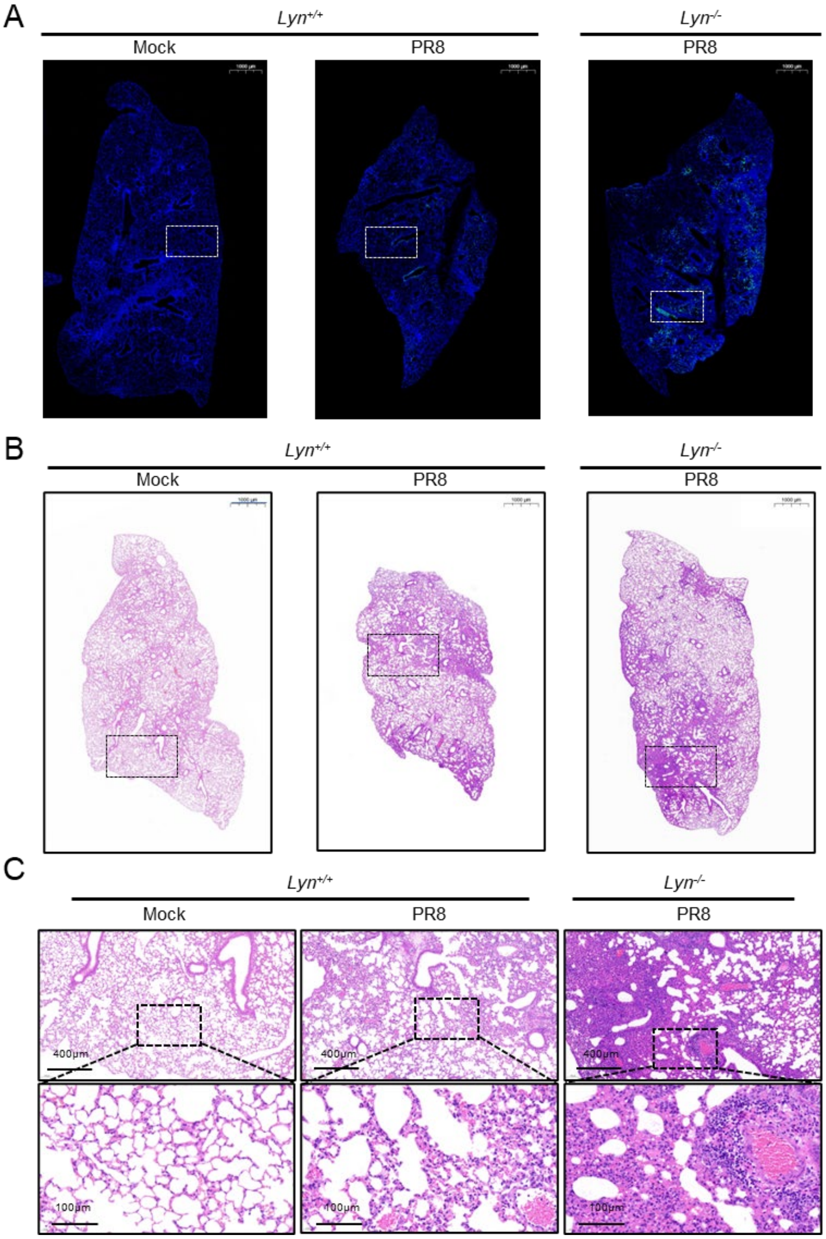

Figure S2. LYN deficiency promotes tissue lesions induced by IAV infection. (A) Viral antigen in the lungs of *Lyn*$^{+/+}$and *Lyn*$^{-/-}$ mice that were euthanized on day 3 post-inoculation with PR8 virus (10^4 EID_{50}) were detected by means of immunofluorescent staining. (B-C) Evaluation of histopathological changes in the lungs of infected and uninfected mice by means of HE staining on day 3 post-inoculation.

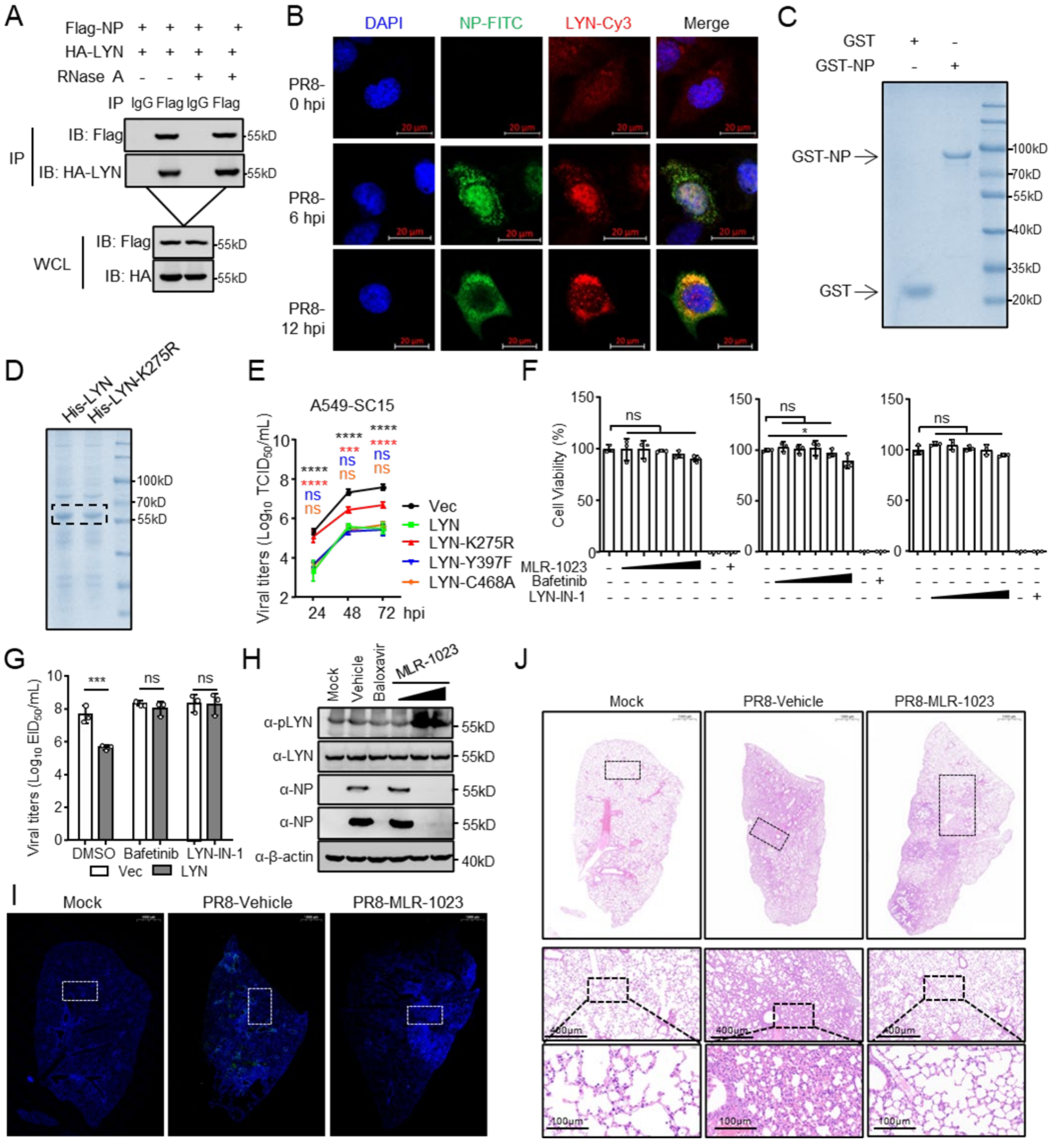

Figure S3. The effect of LYN inhibitor on the replication of IAV. (A) HEK293T cells were transfected with HA-LYN together with Flag-NP for 24 h. Then, the cell lysate was treated with or without RNase A before co-IP and western blotting. (B) U2OS cells were infected with PR8 virus for 0/6/12 h. Then, the cells were fixed with 4% paraformaldehyde and stained with anti-LYN and anti-NP antibody before confocal microscopy. Scale bar: 20 μm. (C) Purified GST and GST-NP were stained by coomassie blue. (D) Purified His-LYN and His-LYN-K275R were stained by coomassie blue and indicated in dashed box. (E) A549 cells were transfected with the expression plasmid of LYN, LYN mutants, or

Vec for 24 h before being infected with SC15 virus (MOI = 0.01). Supernatants were collected at the indicated times post-infection for the $TCID_{50}$ assay. The significant difference between each group and wildtype LYN were labeled in indicated colors. (F) Effect of LYN agonist and inhibitors on cell viability detected by CCK8. (G) A549 cells were transfected with either LYN or Vec for 24 h. Then, the cells were infected with PR8 virus (MOI = 0.01), and treated with Bafetinib (20 μM), LYN-IN-1 (20 μM), or DMSO. At 48 h post-infection, the supernatants were collected for the EID_{50} assay. (H) MLR-1023 (12.5/25/50 mg/kg) were injected intraperitoneally from 7 days before challenge and administered for 11 days, once daily. The commercial anti-IAV drugs baloxavir (10mg/kg) was used as a positive control. At day 3 post-infection with PR8 virus (10^5 EID_{50}), the mice were euthanized, and the lungs were collected for western blotting. (I) Partial lung tissues collected on day 3 post-inoculation were subjected to immunofluorescence. (J) Evaluation of histopathological changes in the lungs of infected and uninfected mice by means of HE staining on day 3 post-inoculation. Statistical significance was determined by a one-way ANOVA in G and I, or two-way ANOVA in F and H.

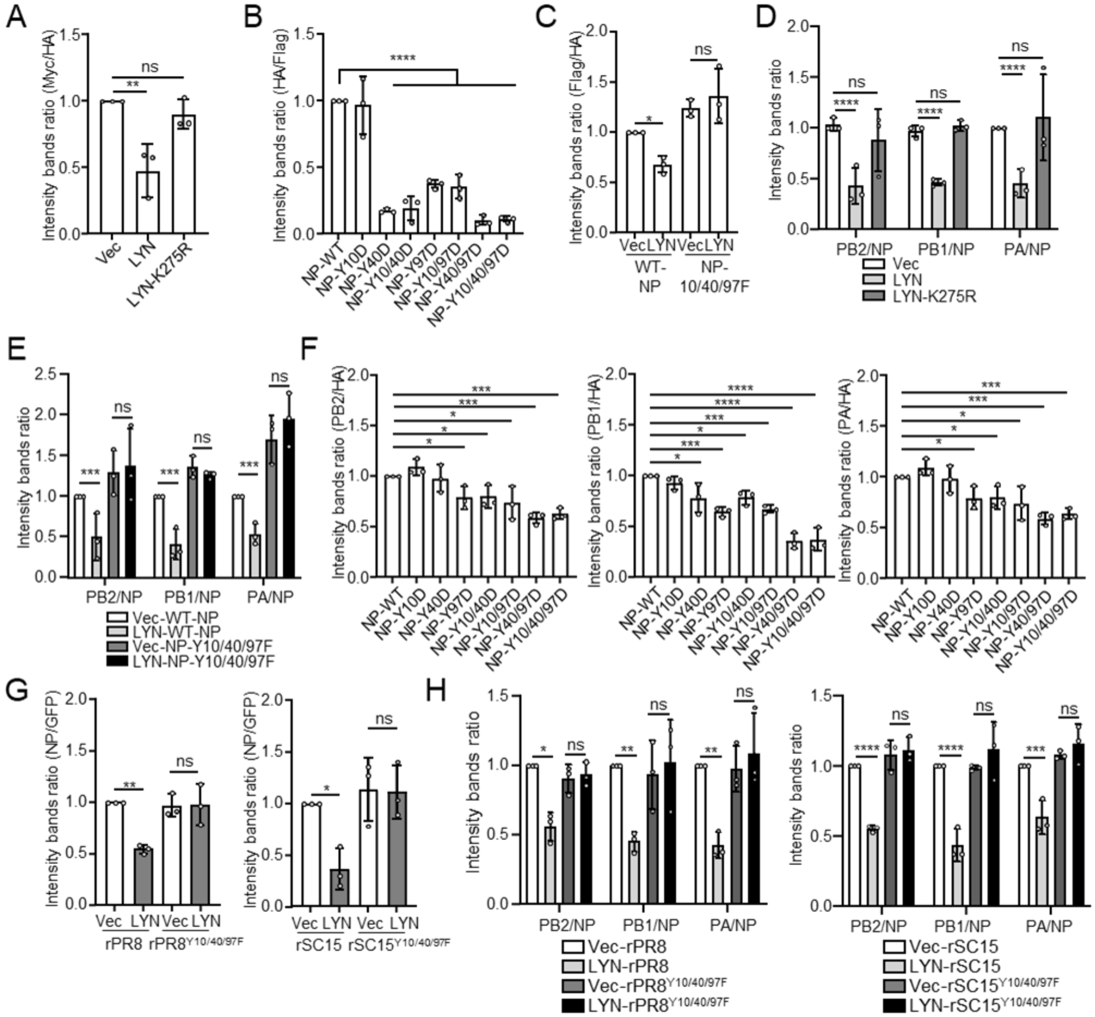

Figure S4. Y10/40/97 phosphorylation of NP inhibits vRNP assembly. (A) The intensity ratio of Myc-NP normalized to HA-NP in IP samples of Fig. 6A were determined using ImageJ. (B) The intensity ratio of HA-NP normalized to Flag-NP in IP samples of Fig. 6C were determined using ImageJ. (C) The intensity ratio of Flag-NP normalized to HA-NP in IP samples of Fig. 6D were determined using ImageJ. (D) The intensity ratio of PB2, PBA, and PA normalized to NP in IP samples of Fig. 6E were determined using ImageJ. (E) The intensity ratio of PB2, PBA, and PA normalized to HA-NP in IP samples of Fig. 6G were determined using ImageJ. (F) The intensity ratio of PB2, PBA, and PA normalized to HA-NP in IP samples of Fig. 6H were determined using ImageJ. (G) The intensity ratio of NP normalized to GFP-NP in IP samples of Fig. 7G were determined using ImageJ. (H) The intensity ratio of PB2, PBA, and PA normalized to NP in IP samples of Fig. 7H were determined using ImageJ. The data shown represent three independent experiments.

Statistical significance was determined by a one-way ANOVA in A, B, C, and F, or two-way ANOVA in D, E, G, and H.

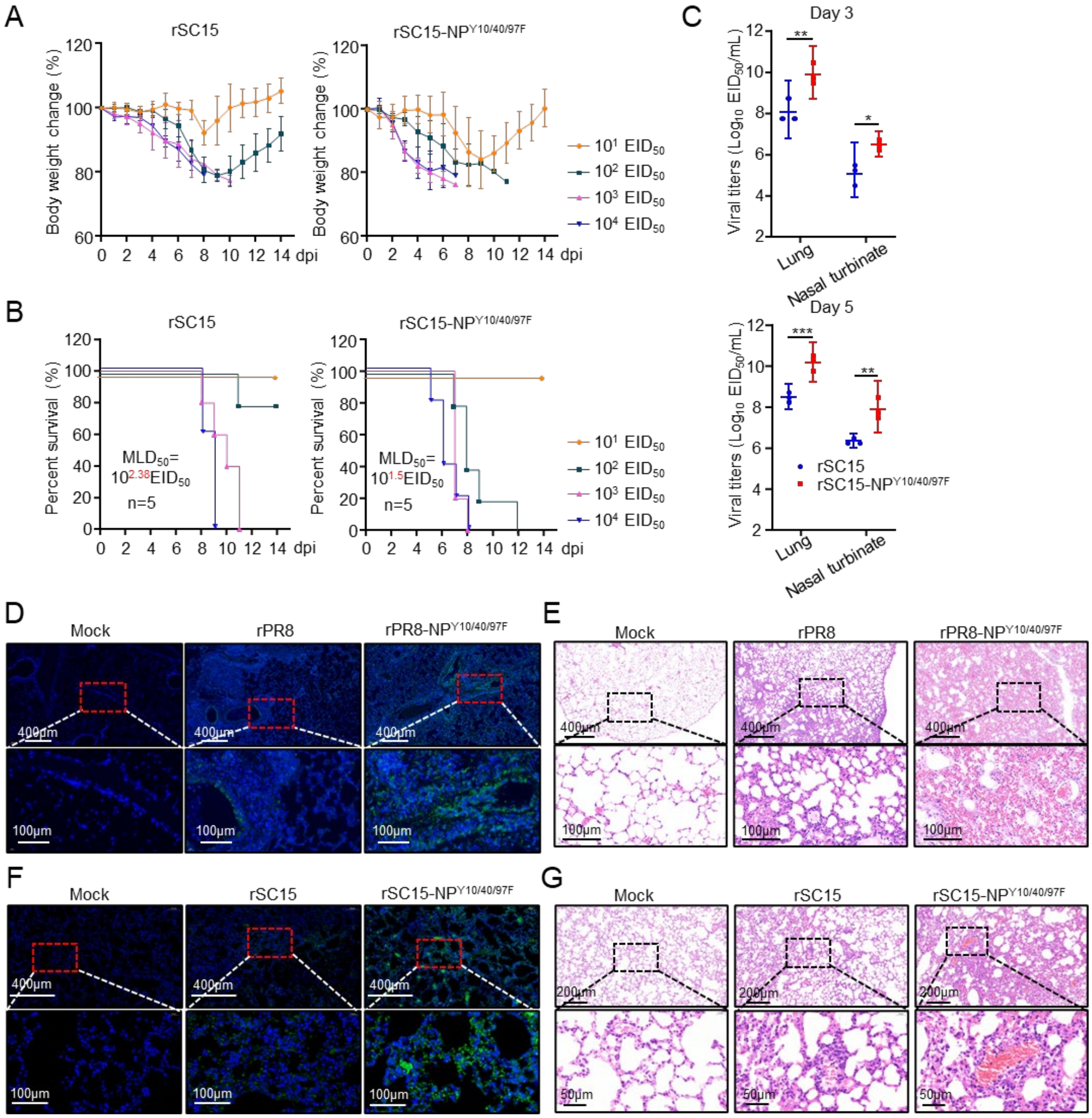

Figure S5. The Y10/40/97F mutation of NP increases the replication and pathogenicity of H5N6 virus *in vivo*. (A-B) Groups of five C57BL/6N mice were intranasally inoculated with 10-fold serial dilutions containing 10^1 to 10^4 EID_{50} of rSC15 or rSC15-$NP^{Y10/40/97F}$ virus. Changes in body weight (A) and survival (B) were monitored for 14 days after virus challenge. The MLD_{50} was calculated by using the method of Reed and Muench. (C) Groups of six C57BL/6N mice intranasally infected with 10^4 EID_{50} of rSC15 or rSC15-$NP^{Y10/40/97F}$ virus were euthanized on days 3 and 5 post-inoculation, and their lungs and nasal turbinate were collected for the EID_{50} assay. The data shown represent three independent experiments. Statistical significance was determined by a two-way

ANOVA. (D) Immunofluorescent staining of lung sections of mice infected with 10^4 EID_{50} of rPR8 or rPR8-$NP^{Y10/40/97F}$ virus at 3 dpi. The viral NP protein was stained green, and the nucleus was stained blue. (E) Evaluation of histopathological changes in the lungs of infected and uninfected mice by means of HE staining at 3 dpi. (F) Immunofluorescent staining of lung sections of mice infected with 10^4 EID_{50} of rSC15 or rSC15-$NP^{Y10/40/97F}$ virus at 3 dpi. The viral NP protein was stained green, and the nucleus was stained blue. (G) Evaluation of histopathological changes in the lungs of infected and uninfected mice by means of HE staining at 3 dpi. Statistical significance was determined by a two-way ANOVA in C.

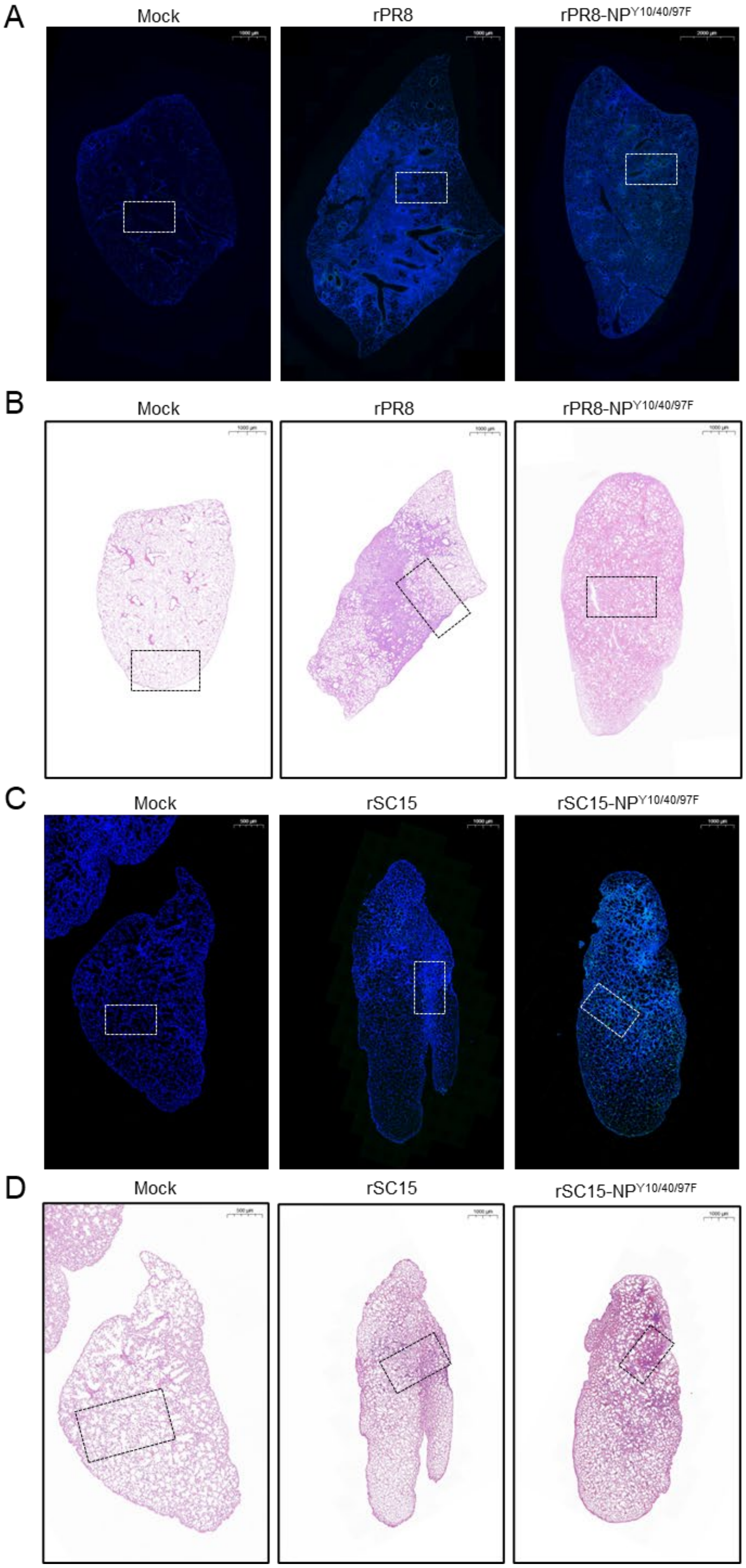

Figure S6. The viral load and histopathological changes of lung tissues from mice infected with wild-type or mutant viruses. (A-B) Immunofluorescent and HE staining of

lung sections from mice infected with 10^4 EID_{50} of rPR8 or rPR8-$NP^{Y10/40/97F}$ virus at 3 dpi. (C-D) Immunofluorescent and HE staining of lung sections from mice infected with 10^4 EID_{50} of rSC15 or rSC15-$NP^{Y10/40/97F}$ virus at 3 dpi.

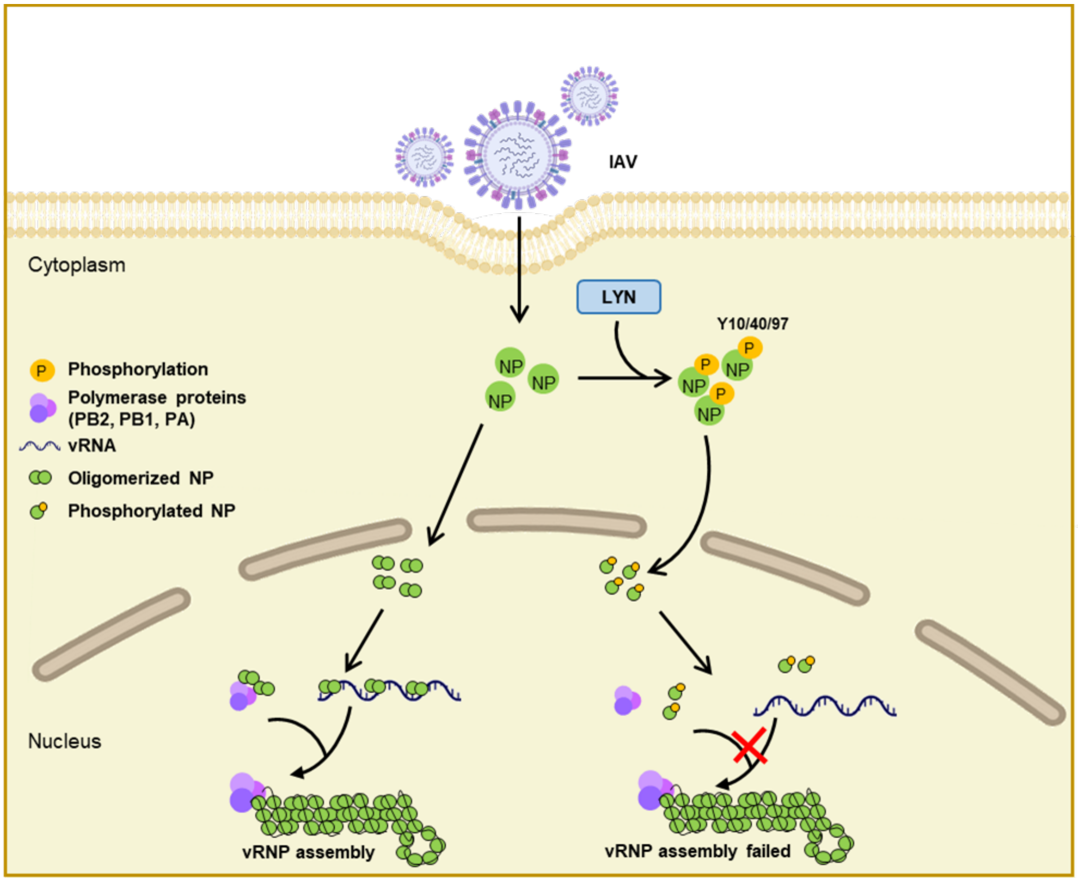

Figure S7. A model of the tyrosine phosphorylation of NP catalyzed by LYN at Y10/40/97 showing restriction to the replication and virulence of IAV.
